# Circ_CEA promotes the interaction between the p53 and cyclin-dependent kinases 1 as a scaffold to inhibit the apoptosis of gastric cancer

**DOI:** 10.1038/s41419-022-05254-1

**Published:** 2022-09-27

**Authors:** Yuan Yuan, Xiaojing Zhang, Kaining Du, Xiaohui Zhu, Shanshan Chang, Yang Chen, Yidan Xu, Jiachun Sun, Xiaonuan Luo, Shiqi Deng, Ying Qin, Xianling Feng, Yanjie Wei, Xinmin Fan, Ziyang Liu, Baixin Zheng, Hassan Ashktorab, Duane Smoot, Song Li, Xiaoxun Xie, Zhe Jin, Yin Peng

**Affiliations:** 1 Guangdong Provincial Key Laboratory of Genome Stability and Disease Prevention and Regional Immunity and Diseases, Department of Pathology, Shenzhen University Health Science Center, Shenzhen, Guangdong 518060 People’s Republic of China; 2 Department of Pharmacology, College of Pharmacy, Shenzhen Technology University, Shenzhen, Guangdong 518118 People’s Republic of China; 3 The First Affiliated Hospital, and College of Clinical Medicine of Henan University of Science and Technology, Henan Key Laboratory of Cancer Epigenetics, No. 24, Jinhua Road, Jianxi District, Luoyang, Henan 471003 People’s Republic of China; 4 Department of Gastrointestinal Surgery, The First Affiliated Hospital of Shenzhen University, Shenzhen Second People’s Hospital, Shenzhen, Guangdong 518000 People’s Republic of China; 5 Center for High Performance Computing, Shenzhen Institutes of Advanced Technology, Shenzhen, Guangdong 518000 People’s Republic of China; 6 Department of Medicine and Cancer Center, Howard University, College of Medicine, Washington, DC 20060 USA; 7 Department of Medicine, Meharry Medical Center, Nashville, TN 37208 USA; 8 Shenzhen Science & Technology Development Exchange Center, Shenzhen, Guangdong 518055 People’s Republic of China; 9 School of Basic Medical Sciences, Guangxi Medical University, Nanning, 530021 Guangxi People’s Republic of China

**Keywords:** Gastric cancer, Oncogenes

## Abstract

Circular RNAs (circRNAs) have been reported to play essential roles in tumorigenesis and progression. This study aimed to identify dysregulated circRNAs in gastric cancer (GC) and investigate the functions and underlying mechanism of these circRNAs in GC development. Here, we identify circ_CEA, a circRNA derived from the back-splicing of CEA cell adhesion molecule 5 (CEA) gene, as a novel oncogenic driver of GC. Circ_CEA is significantly upregulated in GC tissues and cell lines. Circ_CEA knockdown suppresses GC progression, and enhances stress-induced apoptosis in vitro and in vivo. Mechanistically, circ_CEA interacts with p53 and cyclin-dependent kinases 1 (CDK1) proteins. It serves as a scaffold to enhance the association between p53 and CDK1. As a result, circ_CEA promotes CDK1-mediated p53 phosphorylation at Ser315, then decreases p53 nuclear retention and suppresses its activity, leading to the downregulation of p53 target genes associated with apoptosis. These findings suggest that circ_CEA protects GC cells from stress-induced apoptosis, via acting as a protein scaffold and interacting with p53 and CDK1 proteins. Combinational therapy of targeting circ_CEA and chemo-drug caused more cell apoptosis, decreased tumor volume and alleviated side effect induced by chemo-drug. Therefore, targeting circ_CEA might present a novel treatment strategy for GC.

## Introduction

Gastric cancer (GC) is one of the most prevalent carcinomas, with over a million new cases diagnosed worldwide each year [1]. Although its survival rate has significantly increased over the past 40 years, GC remains the third leading cause of oncological death in 2018 [2, 3]. Surgery has been considered the only curative therapeutic strategy for GC and the addition of chemotherapy improves the survival of GC patients effectively [4, 5]. However, traditional chemotherapeutic reagents used for GC therapy, such as Doxorubicin (Dox), exhibit severe side-effects, and the development of drug resistance in patients also limits their clinical application [6–8]. Thus, it is urgent to investigate the mechanism of GC pathogenesis and find new targets for the diagnosis and treatment of GC.

Circular RNAs (circRNA), a group of endogenous RNA molecules with unique closed cyclic structures, have high stability and tissue-specific/developmental-stage-specific expression pattern [9–12]. Recently, the dysregulation of circRNAs have been found in various tumors, including basal cell carcinoma [13, 14], colorectal cancer [15, 16], hepatocellular carcinoma [17, 18], and GC [19], indicating their crucial roles in cancer development. circRNAs exhibit multiple functions in cancer pathogenesis and progression, such as functioning as microRNA (miRNA) sponges [20, 21] or serving as templates for protein synthesis [22, 23]. In the previous study, we showed that circAXIN1 promotes GC development via encoding a novel oncogenic protein, AXIN1-295aa [24]. Furtherly, recent studies have reported that some circRNA act as protein scaffolds to affect the interaction between two or more proteins [25, 26]. Liu et al. described a novel circRNA in esophageal cancer, cDOPEY2, which functions as a protein scaffold to promote the interaction between TRIM25, an E3 ligase, and cytoplasmic polyadenylation element binding protein (CPEB4), leading to the ubiquitination and degradation of CPEB4 [25]. However, the exact mechanisms by which circRNAs act as protein scaffolds and how this function of circRNA affects tumorigenesis and progression of GC remain unclear.

p53, a key tumor suppressor, participates in cancer development via regulating cell cycle, apoptosis, and metabolism [27]. p53 mutation is observed in more than 50% of all human cancers, including GC [28, 29]. Its expression and mutation status are associated with GC progression and prognosis [30, 31]. Furtherly, recent studies have shown that some circRNAs are involved in regulating the expression [32], stability [33, 34], and activity of p53 [35]. CircRNA CDR1as has been found to directly bind to p53 and then disrupt the p53/MDM2 complex, resulting in the inhibition of p53 ubiquitination and degradation [33]. It may also preserve p53 function via forming a protective complex with p53. Gong et al. showed that circEsyt2 regulates p53 pre-mRNA splicing via directly interacting with polyC-binding protein 1, an RNA splicing factor [32]. However, the roles of circRNAs in the regulation of p53 function have not yet been elucidated.

Here, we identified circ_CEA, derived from the CEA cell adhesion molecule 5 (CEA) gene, as a novel oncogenic driver of GC. Circ_CEA interacts with p53 and cyclin-dependent kinases 1 (CDK1) proteins and serves as a scaffold to enhance the association between them. Consequently, it promotes CDK1-mediated p53 phosphorylation at Ser315, suppresses p53 activity, and protects GC cells from stress-induced apoptosis, presenting potential therapeutic opportunities.

## Materials and methods

### Cell lines and tissues

Human normal gastric epithelial cells HFE-145 were provided by Dr. Duane T Smoot, Meharry Medical College. Human GC cell lines (AGS, MKN28, BGC-823, MKN45, and SGC-7901) and 293 T (human embryonic kidney) cells were purchased from Cell Bank of the Chinese Academy of Sciences (Shanghai, China). All cell lines were maintained in DMEM medium (high glucose) (Hyclone, Utah, USA), supplemented with 10% FBS (Gibco) in a humidified atmosphere of 5% CO_2_ at 37 °C. All cell lines were tested for mycoplasma regularly and tested for STR profiling. Fresh GC and corresponding adjacent tissues were obtained from Shenzhen Second People’s Hospital (Shenzhen, China).

### Plasmid and siRNA of circ_CEA and cell transfection

A circ-CEA overexpressing plasmid was constructed by Geneseed Biotechnology Corporation (Guangzhou, China). A nucleotide fragment containing the full length of circ_CEA cDNA and flanking sequences (short intronic repeat sequences) [36] was synthesized and cloned into vector pCDH-CMV-MCS-EF1-Puro. The empty vector was used as a negative control. The plasmids were transfected into cells using Lipofectamine™ 3000 Transfection Reagent (Invitrogen, Carlsbad, California, USA).

siRNAs for circ_CEA were designed according to the junction site sequence. siNC was used as a negative control. The sequences of these siRNAs were shown as follows: siRNA#1: CAGGAAGACTGATGGGCCG; siRNA#2: GGAAGACTGATGGGCCGGA; siRNA#3: AAGACTGATGGGCCGGACA; siNC: TTCTCCGAACGTGTCACGT. The siRNAs (60 nM) were transfected into cells using Lipofectamine RNAi MAX (Invitrogen).

### RNA-seq assay

Total RNA was isolated from 5 clinical GCs and corresponding adjacent tissues. Then, ribosomal RNAs were removed using a Ribo-Zero^TM^ rRNA removal kit (Illumina, California, USA), followed by linear RNAs depletion via RNase R treatment. The RNA-seq library was constructed using fragmented RNAs as templates, and then sequenced using an Illumina Hiseq 2500 (Chi Biotech, Shenzhen, China). The BWA aligner was used to align the RNA-seq reads to the human reference genome (GRCh38). Then, the CIRI software was used to identify circRNAs, and genomic annotation of circRNAs was performed using the gene annotation file, corresponding to the human reference genome.

### RNase R treatment

Total RNA (2 μg) isolated from cultured cells were incubated with RNase R (3 U/μg) (Geneseed Biotechnology Corporation) or water at 37 °C for 15 min. Next, the RNase R-mediated degradation of circ_CEA and linear RNAs (CEA mRNA and 18 s rRNA) were evaluated by qRT-PCR.

### Nuclear and cytoplasmic extraction

Nuclear and cytoplasmic RNAs and proteins were isolated with Cytoplasmic and Nuclear RNA Purification Kit (Norgen Biotek corp., Canada) and NE-PER^TM^ Nuclear and cytoplasmic extraction reagents (Thermo, USA), respectively.

### Quantitative reverse transcription PCR (qRT-PCR)

Total RNA was isolated from cells or clinical samples using TRIzol Reagent (Invitrogen). Next, cDNA synthesis and real-time PCR were conducted using GoScript^TM^ Reverse Transcription Mix (Promega, USA) and GoTaq® qPCR Master Mix (Promega, USA), respectively. The primers for qRT-PCR were listed in Supplementary Table 1. GAPDH and 18 s were used for normalization. The results were analyzed by 2^−ΔΔCt^ method.

### Fluorescence in situ hybridization (FISH) and immunofluorescence (IF)

An FITC labeled oligonucleotide probe for circ_CEA (5′-FITC-tgtccggcccatcagtcttcct-3′ FITC) was synthesized by Geneseed Biotechnology Corporation (Guangzhou, China). The procedure is performed as previously described [24]. AGS cells were fixed and antigen was retrieved by autoclaving. The slide was washed and dehydrated. Prehybridization, hybridization, and post-hybridization were performed. After in situ hybridization, the slide was blocked and primary antibody was added, and the sections were incubated overnight at 4 °C in a humid chamber. The slide was rinsed and then incubated with a secondary antibody. The nucleus was stained with 4’,6-diamidino-2-phenylindole (DAPI). Images were captured using the confocal microscope.

### Western blotting

Protein extraction was conducted using RIPA buffer (Sigma-Aldrich, USA) supplemented with protease inhibitor cocktail Tablets (Roche, Switzerland) and Pierce^TM^ phosphatase inhibitor (Thermo Scientific, USA). The antibodies against p53 (#2524), p-p53 ser315 (#2528), Forkhead box protein O3 (FoxO3) (#2497), CDK1(#9116), B-cell lymphoma-2 (Bcl-2) family proteins (#98322, #9941), poly (ADP-ribose) polymerase (PARP) (#9542), c-PARP (#5625) and glyceraldehyde-3-phosphate dehydrogenase (GAPDH) (1:1000, Cell Signaling Technology, Beverly, MA, USA) were used.

### In vitro functional assays

To evaluate cellular proliferation, EdU assay was conducted using Cell-Light^TM^ EdU Apollo567 In Vitro Kit (RIBOBIO, Guangzhou, China), following the manufacturer’s instructions.

Cellular migration was evaluated by wound-healing assay. Cells were seeded in six-well plates. After 48 hr of siRNA transfection, straight scratches were made using pipette tips, and then the progression of wound-healing was recorded via measuring wound width at 0, 24, and 48 hr following scratching.

Transwell migration assay was conducted using a 24-well transwell chamber (BD, San Diego, USA). siRNA-transfected cells (1 × 10^5^) were cultured in the upper chamber, with a serum-free medium, while the medium containing 20% FBS was added into the lower chamber. After 24 hr of culture, the cells were fixed in 4% paraformaldehyde and stained with hematoxylin. Finally, the cells that migrated through the filter membrane were counted, under a microscope.

To evaluate the colony formation ability of cells, siRNA-transfected cells (200) were uniformly dispersed and seeded in a 24-well plate. After 2 weeks of culture, the cells were fixed in 4% paraformaldehyde and stained with crystal violet solution. The cell colonies were counted under a microscope.

To evaluate apoptosis, Annexin V/PI and Hoechst 33342/PI staining were conducted by using FITC Annexin V/dead cell apoptosis kit (Invitrogen) and Hoechst 33342/PI double stain kit (Solarbio, China), respectively, following the manufacturer’s instructions.

### P53 transcription factor assay

To determine p53 DNA binding activity, siRNA-transfected cells were treated with Dox (1.25 μM) for 1 hr, then p53 transcription factor assay was conducted with TransAM^TM^ p53 kit (Active Motif), following the manufacturer’s instructions.

### MS2/MS2-CP system-based circRNA pull-down

The MS2/MS2-CP-based circRNA pull-down assay and protein mass spectrometry analysis were conducted by Geneseed Biotechnology Corporation. Briefly, a circ_CEA expression vector containing MS2 tag and an expression vector for MS2 coat protein (MS2-CP) were constructed. 293 T cells were divided into four groups, and transfected with the following vectors, respectively: 1. Circ_CEA-MS2 + MS2-CP; 2. Circ_CEA + MS2-CP; 3. Circ_CEA-MS2 + NC; 4. Circ_CEA + NC (NC: a blank control vector for MS2-CP vector). The group 2, 3, and 4 were negative control groups. Then, circRNA pull-down was performed with the antibody against MS2-CP protein or IgG. The immunoprecipitated products were collected and protein mass spectrometry was conducted to identify potential binding proteins of circ_CEA.

### RNA pull-down assay with biotinylated probes for circ_CEA

Cultured cells (10^7^) were lysed in 500 μl co-IP Buffer (Cell lysis buffer for western and IP, Beyotime, China), supplemented with protease inhibitor cocktail Tablets (Roche, Switzerland), Pierce^TM^ phosphatase inhibitor (Thermo Scientific), and Protector RNase Inhibitor (Roche). Next, the cell lysates were incubated with 800 pmol of biotinylated DNA probes for circ_CEA (5′-GCCCATCAGTCTTCCTGAAA-3′) or scramble probes (5′-ATCTAATAGCTCCACGTGCC-3′) at 4 °C overnight. Next, Streptavidin C1 magnetic beads (Invitrogen) were blocked with 2 mg/mL BSA at room temperature for 1 hr, and then added to each binding reaction and incubated at room temperature for 1 hr. After washing in Co-IP Buffer, beads were incubated with Non-Reducing Lane Marker Sample Buffer (Thermo Scientific) at room temperature for 10 min to elute the bound proteins. The proteins were detected by western blotting with the antibodies against p-p53 ser315, p53, CDK1, and FoxO3 (Cell Signaling Technology).

### RNA binding protein immunoprecipitation assay (RIP)

Pierce^TM^ Classic Magnetic IP/Co-IP kit (Thermo Scientific) was used for RIP assay. Cultured cells (10^7^) were lysed in 800 μl Pierce IP Lysis/Wash Buffer supplemented with protease inhibitor cocktail Tablets (Roche), and Protector RNase Inhibitor (Roche). The cell lysate was incubated with antibodies against p53 (#9282), CDK1, or FoxO3 (Cell Signaling Technology) at 4 °C overnight. Mouse IgG1 Isotype control and rabbit mAb IgG XP Isotype control (Cell Signaling Technology) were used as a negative control. Next, 0.25 mg of Pierce Protein A/G Magnetic Beads were added to each sample and incubated at room temperature for 1 hr. Then the pellets were collected, washed with Pierce IP Lysis/Wash Buffer, and then resuspended in TRIzol Reagent (Invitrogen). RNAs were isolated from the pellets and circ_CEA enrichment was evaluated by qRT-PCR assay.

### Co-immunoprecipitation assay

Co-IP assay was conducted with Pierce Classic Magnetic IP/Co-IP Kit (Thermo Scientific). Cultured cells (10^7^) were lysed in 800 μl Pierce IP Lysis/Wash Buffer supplemented with protease inhibitor cocktail Tablets (Roche). The cell lysate was incubated with the anti-CDK1 antibody (Cell Signaling Technology) at 4 °C overnight. Mouse IgG1 Isotype control (Cell Signaling Technology) was used as a negative control. Next, 0.25 mg of Pierce Protein A/G Magnetic Beads were added to each sample and incubated at room temperature for 1 hr. Then the pellets were collected, washed with Pierce IP Lysis/Wash Buffer, and then incubated with Non-Reducing Lane Marker Sample Buffer (Thermo Scientific) at room temperature for 10 min to elute the bound proteins. The p53 proteins co-precipitated with anti-CDK1 antibody were detected by western blotting. To avoid the detection of IgG heavy and light chains, VeriBlot for IP detection reagents (Abcam) were used as secondary antibodies.

### Animal experiments

All animal experiments were conducted in compliance with the Guidelines of Institutional Animal Care and Use Committee of Shenzhen University. Four-week-old female BALB/c nude mice were purchased from Charles River Laboratories (Beijing, China). Cholesterol-conjugated circ_CEA siRNAs and siNC were obtained from Geneseed Biotechnology Corporation. To evaluate the effect of circ_CEA on tumor growth, subcutaneous xenografts were generated by injecting AGS cells (5 × 10^6^ cells/mouse) into the upper back of the mice. After one week of injection, the mice were randomly divided into four groups (*n* = 8 mice in each group): 1. control, 2. circ_CEA simix, 3. Dox and 4. circ_CEA simix + Dox group, and administrated with siNC (10nmol/mouse, intratumoral injection), circ_CEA simix (10 nmol/mouse, intratumoral injection), Dox (2.5 mg/Kg, intraperitoneal injection) +siNC and circ_CEA simix + Dox, respectively, twice a week for 3 weeks. The tumor volumes were measured twice a week and calculated by using the formula: tumor volume = (length × width^2^)/2. The mice were killed and tumor tissues were kept in RNAlater or formaldehyde solution for subsequent experiments.

For in vivo metastasis assay, AGS cells (2.5 × 10^6^ cells/mouse) were injected into the nude mice via tail vein. Then, the mice were randomly divided into four groups (*n* = 6 mice in each group) and administrated with the reagents as described above, twice a week for five weeks. Then, the mice were sacrificed, and the lung and liver tissues were kept in formaldehyde solution. Paraffin sections (4-μm thick) were prepared, and hematoxylin-eosin staining and immunohistochemistry were conducted. The lung metastatic nodules were counted under a microscope.

### Terminal deoxynucleotidyl transferase dUTP nick end labeling (TUNEL) assay

Paraffin sections (4 μm thick) were prepared from subcutaneous tumor samples. TUNEL staining was conducted using Fluorescein (FITC) Tunel Cell Apoptosis Detection Kit (Servicebio, Wuhan, China). The cellular nuclei were stained with DAPI.

### Immunohistochemistry (IHC)

Paraffin sections were prepared from subcutaneous xenografts and immunohistochemical staining was conducted by using the antibodies against Bax (servicebio, Wuhan, China) and cleaved PARP (#5625, Cell Signaling Technology, Beverly, MA, USA). Nuclei were stained by DAPI. The detailed procedure is described previously [24]. In order to evaluate the staining, the staining intensity was scored as 0 (negative), 1 (weak), 2 (moderate) and 3 (strong). In addition, the percentage of positive cells was evaluated and classified as 0 (≤5% positive cells), 1 (6–25%), 2 (26–50%) and 3(≥51%). The IHC score was calculated by multiplying the staining intensity by percentage of positive cells.

### Statistical analysis

To determine the statistical significance of difference, statistical analyses were conducted by student’s *t* test or one-way AVONA. The expression of circ_CEA and CEA correlation study was performed using Pearson correlation analysis. The correlation between the expression level of circ_CEA and pathological factors was analyzed using *χ*^2^ test. The Kaplan–Meier (KM) method is used to analyze the relationship between patient overall survival and CEA expression. A two-tailed *P* value <0.05 was considered statistically significant. The data were presented as mean ± standard deviation (SD).

## Results

### Circ_CEA is upregulated in GC tissues and cell lines

**Fig. 1:**
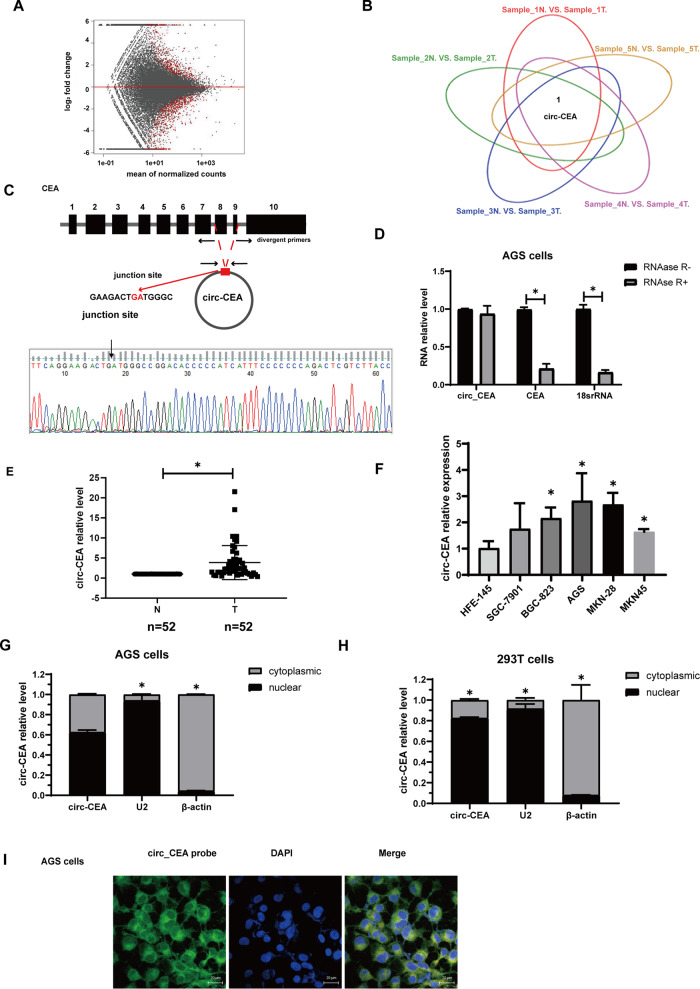
circ_CEA is upregulated in GC tissues and cell lines. **A** MA plots shows significantly dysregulated circRNAs (red points) in GC tissues, compared with the corresponding non-tumoral tissues. *x* axis: mean values of normalized circRNA expression levels in all samples; *y* axis: log2 fold change of circRNA expression levels between GC and non-tumoral tissues. **B** Circ_CEA is the only significantly dysregulated circular RNA in all five paired gastric tissues. **C** Illustration of the genomic location and junction site of circ_CEA. **D** The levels of circ_CEA and linear RNA (CEA mRNA and 18 s rRNA) were evaluated by qRT-PCR assay, following treatment with or without RNase R. Divergent primers were used for circRNA detection, while convergent primers were used for linear RNA detection. *P < 0.05 vs corresponding samples without RNase R treatment. **E** The expression level of circ_CEA in 52 paired GC and non-tumoral tissues was determined by qRT-PCR assay. **F** The expression level of circ_CEA in a normal human gastric epithelial cell line (HFE-145) and gastric cancer cell lines (SGC-7901, BGC-823, AGS, MKN28 and MKN45) was determined by qRT-PCR. **G**, **H** The nuclear and cytoplasmic distribution of circ_CEA in AGS and 293 T cells was evaluated by qRT-PCR assay. U2 and β-actin were used as positive controls in nuclear and cytoplasmic fractions, respectively. *P < 0.05 vs the RNA level in nuclear fraction. **I** FISH using probe of circ_CEA showed that circ_CEA is distributed both in the nucleus and cytoplasm in AGS cells. N non-tumoral tissues, T tumoral tissues. Data are shown as mean±standard deviation (SD).

To investigate potential dysregulated circRNAs in GC, high-throughput circRNA sequencing with ribosomal RNA depletion was conducted using five paired GC and non-tumoral tissues [24, 37]. A total of 478 dysregulated circRNAs (fold change ≥2, *P* < 0.05) were identified, suggesting that these circRNAs may participate in GC development (Fig. 1A). We further analyzed circRNA expression profile in every paired gastric tissue to identify the overlapped dysregulated circRNAs (Fig. 1B). Circ_CEA (circBase ID: hsa_circ_0051240) was found to be the only circRNA which is significantly upregulated in all five GC tissues. Therefore, this circRNA was selected for further study. Circ_CEA (374 nt) is derived from the back-splicing and covalent joining of exon 8 and 9 of CEA gene (Fig. 1C). To characterize circ_CEA, junction-specific divergent primers for circ_CEA and convergent primers for its linear counterpart were designed. Sanger Sequencing was conducted and the junction site of circ_CEA was detected (Fig. 1C). To demonstrate the structure of circ_CEA, we compared RNase R-mediated degradation of circ_CEA and linear RNA. RNase R treatment induced marked degradation of linear RNAs (CEA mRNA and 18 s rRNA) but did not affect circ_CEA level significantly (Fig. 1D). These results indicate a covalent closed structure of circ_CEA. We further evaluated the expression levels of circ_CEA in GC tissues. qRT-PCR assay with 52 GC and corresponding non-tumoral tissues showed that Circ_CEA was significantly upregulated in GC tissues (Fig. 1E). Then, the correlation between circ_CEA expression and clinical pathologic features were determined, using 40 paired GC and non-tumoral tissues (Table 1). The samples were divided into 2 groups, according to circ_CEA expression level. There was a significant correlation between circ_CEA expression and the degree of tumor differentiation. More poorly-differentiated GC tissues were observed in circ_CEA-high group. While circ_CEA level was not associated with gender, age, or lymphatic metastasis. Furtherly, qRT-PCR assay showed that significant upregulation of circ_CEA was observed in GC cell lines (BGC-823, AGS, MKN28 cells, and MKN45) compared to normal HFE-145 cells (Fig. 1F). We have performed the expression correlation analysis between circ_CEA and CEA using RNA-seq data from the Gene Expression Omnibus (GEO) database under accession number GSE152309. It showed that circ_CEA is positively associated with CEA in 10 gastric tissues (Supplementary Fig. 1A). We also performed survival analysis based on CEA expression using KM plotter [38]. The result revealed that CEA is a negative prognosis factor for GC (Supplementary Fig. 1B). Taken together, circ_CEA may be a poor prognostic factor for GC overall survival. In addition, the nuclear and cytoplasmic distribution of circ_CEA in AGS (Fig. 1G, I) and 293 T cells (Fig. 1H) was determined. The results from FISH assay using circ_CEA probe and PCR following subcellular fractionation revealed that circ_CEA is distributed both in the nucleus and cytoplasm in AGS and 293 T cells.

**Table 1:** The correlation between the expression level of circ_CEA and the clinicopathologic characteristics of GC (*n* = 40).

	Circ_CEA expression		*P*
	High	Low	
GC tissues	20	20	
Gender			0.324
male	15	12	
Female	5	8	
Age			0.687
≤50	4	3	
>50	16	17	
Lymphatic metastasis			0.744
present	14	13	
absent	6	7	
Differentiation			0.011*
Well	1	4	
Moderate	9	15	
Poor	10	1	

^*^P < 0.05.

### Circ_CEA enhances progression of GC, and suppresses stress-induced apoptosis in GC cells

**Fig. 2:**
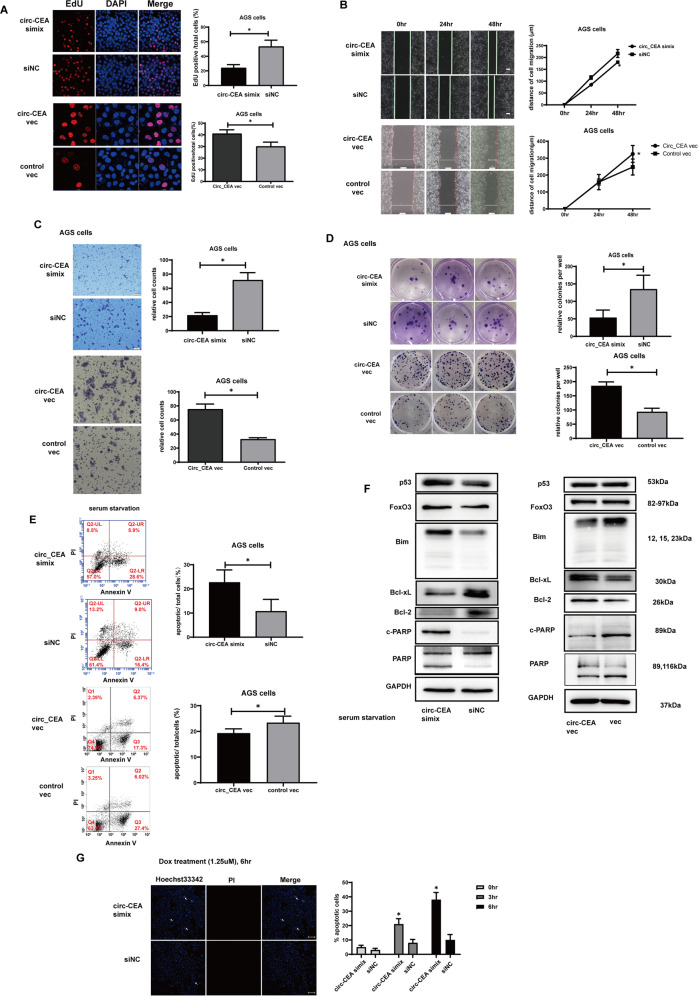
circ_CEA enhances progression of GC, and suppresses stress-induced apoptosis in GC cells. **A** Cellular proliferation was evaluated by EdU assay in AGS transfected with circ_CEA simix or circ_CEA overexpression vector. **B** Cellular migration was determined via wound-healing assay in AGS cells transfected with circ_CEA simix or circ_CEA overexpression vector. **C** Cellular migration was further evaluated by transwell migration assay in AGS cells transfected with circ_CEA simix or circ_CEA overexpression vector. **D** Colony formation assay was performed in AGS cells following the transfection of circ_CEA simix or circ_CEA overexpression vector. **E** AGS cells was transfected with circ_CEA simix or circ_CEA overexpression vector, followed by serum starvation treatment for 72 hr. The serum starvation-induced apoptosis was evaluated via Annexin V/ PI staining, and apoptotic cells were measured by flow cytometry. **F** After serum starvation for 72 hr, the expression levels of apoptosis-associated proteins were evaluated by western blotting assay in AGS cells transfected with circ_CEA simix or circ_CEA overexpression vector. **G** AGS cells were transfected with circ_CEA simix or siNC, followed by Dox treatment (1.25 μM) for the indicated time. The Dox-induced apoptosis was evaluated via hoechst 33342/PI staining. White arrows indicate Hoechst 33342 positive/PI-negative cells (apoptotic cells). *P < 0.05 vs the percentage of apoptotic cells in corresponding siNC-transfected cells. Data are represented as mean± standard deviation (SD).

Three siRNAs (siRNA#1, 2, 3) specifically targeting the junction site of circ_CEA were designed (Supplementary Fig. 2A). The siRNA#2, siRNA#3, and simix (the mixture of siRNA#2 and siRNA#3) exhibited satisfactory knockdown efficiencies, while these circ_CEA siRNAs did not affect the mRNA expression of its host gene, CEA. The simix was used in the subsequent experiments. In addition, circ_CEA overexpression vectors were transfected into AGS cells, and the significant upregulation of circ_CEA was observed (Supplementary Fig. 2B). To investigate the roles of circ_CEA in GC progression, function assays were performed in vitro. EdU assay showed that circ_CEA knockdown significantly reduced the proliferation of AGS cells, while circ_CEA overexpression promoted the proliferation of AGS cells (Fig. 2A). Additionally, the migration abilities of GC cells were evaluated by wound-healing assay and transwell migration assay in AGS cells. A significant decrease in cellular migration was observed following ablation of circ_CEA and an enhancement of cellular migration was detected after circ_CEA overexpression in AGS cells (Fig. 2B, C). Similar results were observed in BGC-823 cells and MKN45 cells (Supplementary Fig. 2C–E). Furthermore, the colony formation ability of AGS cells was attenuated following circ_CEA knockdown, but promoted after circ_CEA overexpression (Fig. 2D).

The function of circRNA is usually associated with the host gene function. It has been reported that CEA is related with anoikis which is a form of apoptosis [39]. This gave us hints to study the relation between circ_CEA and apoptosis. To investigate the effect of circ_CEA on stress-induced apoptosis, AGS cells with circ_CEA knockdown or overexpression were subjected to serum starvation for 72 hr, and then apoptosis was evaluated via flow cytometry using annexin V/PI staining (Fig. 2E). Serum starvation-induced early apoptosis was enhanced following circ_CEA knockdown, but inhibited after circ_CEA overexpression. Furtherly, the expression levels of apoptosis-associated proteins were determined by Western Blotting assay, after serum starvation treatment. The expression levels of p53 and FoxO3 proteins were increased in circ_CEA siRNA-transfected cells (Fig. 2F). Circ_CEA downregulation increased the level of pro-apoptotic Bcl-2 interacting mediator of cell death (Bim) protein but decreased the levels of anti-apoptotic B-cell lymphoma-extra large (Bcl-xL) and Bcl-2 proteins in AGS and BGC-823 cells (Fig. 2F and Supplementary Fig. 2F). In addition, increased poly(ADP-ribose) polymerase (PARP) cleavage was observed in siRNA-transfected cells, indicating a significant increase in apoptosis (Fig. 2F and Supplementary Fig. 2F). Furtherly, circ_CEA overexpression did not affect the expression of p53 and FoxO3 significantly. Its upregulation significantly suppressed the expression of pro-apoptotic Bim and PARP cleavage, but promoted the expression of anti-apoptotic Bcl-xL and Bcl-2 in AGS cells. These findings showed that circ_CEA suppressed serum starvation-induced apoptosis in AGS and BGC-823 cells. We then investigated the effect of circ_CEA on Dox-induced apoptosis. Dox, an anthracycline antibiotic, induces cell death via DNA topoisomerase II-mediated DNA damage, and is wildly used in the treatment of various types of cancer [40]. Hoechst 33342/PI staining was performed to evaluate Dox-induced apoptosis. After Dox treatment, circ_CEA siRNA transfection significantly increased apoptotic (Hoechst 33342 positive/ PI negative) cells, suggesting that circ_CEA downregulation enhanced Dox-induced apoptosis in AGS cells (Fig. 2G). Therefore, our findings showed that circ_CEA exhibits oncogenic properties in GC cells via enhancing cell proliferation, migration, and colony formation, but suppressing stress-induced apoptosis in GC cells.

### Circ_CEA interacts with p53 and CDK1 proteins

**Fig. 3:**
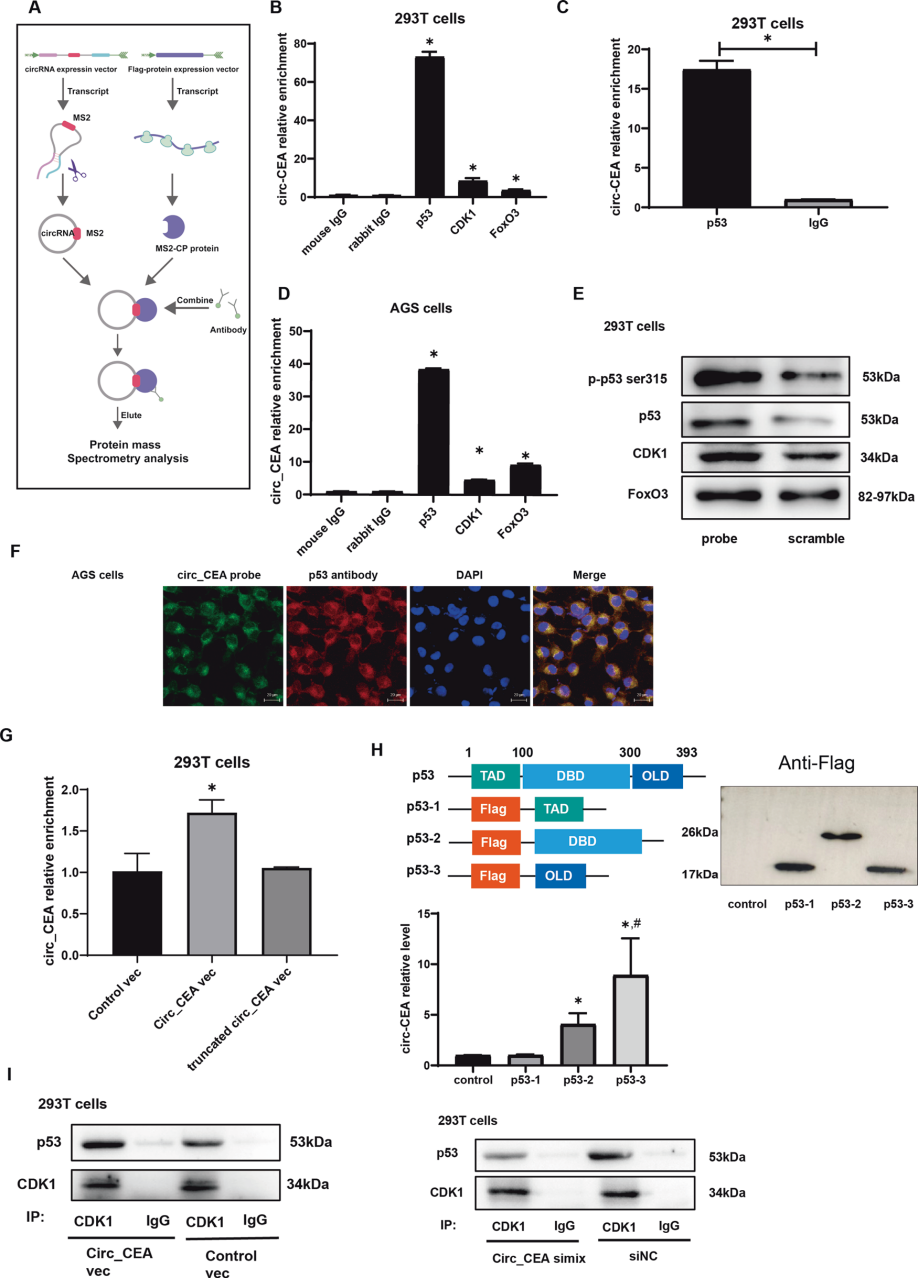
circ_CEA interacts with p53 and CDK1 proteins. **A** Schematic illustration of MS2/MS2-CP based RNA pull-down assay. **B** RNA binding protein immunoprecipitation (RIP) assay was performed by using the antibodies against p53, CDK1, and FoxO3 in 293 T cells transfected with circ_CEA expression vector. Mouse IgG and rabbit IgG were used as negative control. *P < 0.05 vs corresponding negative control. **C** RIP assay was conducted by using anti-p53 antibody in 293 T cells. **D** RIP assay was conducted using anti-p53, CDK1, and FoxO3 antibodies in AGS cells transfected with circ_CEA expression vector. Mouse IgG and rabbit IgG were used as negative control. *P < 0.05 vs corresponding negative control. **E** AGS cells were transfected with circ_CEA expression vector, and RNA pull-down assay was performed by using a biotinylated oligo probe targeting the junction site of circ_CEA. The proteins co-precipitated with circ_CEA were detected by Western Blotting, using the antibodies against p-p53 ser315, p53, CDK1 and FoxO3. A scramble probe was used as a negative control. **F** FISH of circ_CEA and immunostaining of p53 results illustrated the colocalization of circ_CEA and p53. **G** Sequence (180–290) of circ_CEA is essential for the interaction between circ_CEA and p53. A plasmid encoding a truncated circ_CEA in which the sequence (180–290) was deleted was constructed and RIP experiments were performed by using an anti-p53 antibody. **H** OLD of p53 shows a higher binding efficiency to circ_CEA. Plasmids encoding different truncated Flag-tagged p53 were constructed. RIP experiments were performed by using anti-flag antibodies. Data are represented as mean± standard deviation (SD). *P < 0.05 vs control; ^#^*P* < 0.05 vs p53-2. **i** Co_IP assay was conducted with the anti- CDK1 antibody in 293 T cells transfected with circ_CEA expression vector or siRNA. The p53 proteins co-immunoprecipitated by the anti-CDK1 antibody were detected by western blotting.

We next investigated the mechanism by which circ_CEA suppresses stress-induced apoptosis in GC cells. We hypothesized that circ_CEA is involved in apoptosis potentially via regulating the expression of apoptosis-associated proteins, or interacting with these proteins. To identify potential proteins interacting with circ_CEA, we performed an MS2/MS2-CP based RNA pull-down assay (Fig. 3A). A circ_CEA expression vector containing MS2 tag, which is an RNA aptamer derived from bacteriophage, was constructed [41, 42]. Meanwhile, another expression plasmid for MS2 coat protein (MS2-CP), which can specially bind to the MS2 tag, was also constructed. After the co-transfection of these two plasmids into 293 T cells, the circ_CEA with MS2 tag was transcribed and circularized and interacted with its protein partners in the cells. Meanwhile the MS2-CP protein was expressed and specifically bound to the MS2 tag. Then immunoprecipitation assay was performed using the antibody against the MS2-CP protein. The precipitation products were collected, and protein mass spectrometry analysis was conducted. We identified 94 co-immunoprecipitated proteins (Supplementary Table 2). Next, KEGG enrichment analysis showed that these proteins were mainly associated with endocytosis, spliceosome, cell cycle, and p53 signaling pathway (Supplementary Fig. 3A). Among these proteins, p53 and CDK1 associated with p53 signaling pathway have caught our attention. P53 is activated in response to various stress stimuli, and acts as a tumor suppress via inducing cellular apoptosis [43]. To further confirm whether circ_CEA interacts with p53 and CDK1 proteins, RIP assay was conducted in 293 T and AGS cells (Fig. 3B–D). We also investigated the interaction between circ_CEA and FoxO3, an important transcriptional factor involved in cellular stress response and apoptosis [44]. Circ_CEA was co-precipitated by anti-p53 antibody in 293 T cells with (Fig. 3B) or without circ_CEA overexpression (Fig. 3C). It was also co-precipitated by anti-CDK1 or FoxO3 antibody in 293 T cells transfected with circ_CEA expression plasmid (Fig. 3B). Consistent with the findings in 293 T cells, circ_CEA was co-precipitated by the antibodies against p53, CDK1 and FoxO3 in AGS cells transfected with circ_CEA expression vector (Fig. 3D). Furtherly, RNA pull-down assay was conducted in 293 T cells transfected with circ_CEA expression vector, using a biotin-labeled oligonucleotide probe, which specifically targeted the junction site of circ_CEA. Higher levels of p-p53 ser315, p53, CDK1, and FoxO3 proteins were precipitated by the circ_CEA probe, compared to the scrambled probe (Fig. 3E). CDK1, which belongs to a family of Ser/Thr protein kinase, has been reported to mediate p53 phosphorylation at ser315 [45]. Furtherly, FISH-IF assay was performed using circ_CEA probe and anti-p53 antibody in AGS cells, and the colocalization of circ_CEA and p53 was confirmed (Fig. 3F). These findings suggested that circ_CEA interacts with p53, CDK1, and FoxO3 proteins. In order to identify the region in p53 and circ_CEA responsible for the interaction with each other, we have performed a computer simulation of the interaction between circ_CEA and p53, and predicted the binding sites via several methods (Supplementary Fig. 3B, C). In our first method, we simulated their interaction in HDOCK website (http://hdock.phys.hust.edu.cn/), by using circ_CEA and p53 sequences. HDOCK server can be used for the prediction of protein-DNA/RNA docking based on a hybrid strategy (Supplementary Fig. 3B). In the second method, we predicted the secondary and tertiary structures of circ_CEA using RNAfold (Supplementary Fig. 3C). Then, we obtained the tertiary structures of p53 from Protein Data Bank (PDB). Furtherly, we simulated circ_CEA and p53 interaction in HDOCK, by using their tertiary structures. In the third method, we simulated their interaction by using PRIdictor server (PRIdictor (inha.ac.kr)) [46]. Based on the results of these methods, we predicted that a segment of circ_CEA (180–290) may be essential for the interaction between circ_CEA and p53. To investigate whether this sequence is responsible for the binding of p53, we constructed a plasmid encoding a truncated circ_CEA in which the sequence (180–290) was deleted and performed RIP experiments by using an anti-p53 antibody. The results showed that circ-CEA interacts with p53 through this region (Fig. 3G).

In addition, p53 proteins mainly contain three functional domains: an N-terminal transactivation domain (TAD), a central DNA binding domain (DBD), and a C-terminal oligomerization domain (OLD) (Fig. 3H). Here, three plasmids encoding flag-tagged functional domains of p53 (TAD:1–100; DBD:100–300; OLD:300–393) were constructed. Then, these vectors of truncated p53 were co-transfected with circ_CEA over-expression vectors into 293 T cells, and RIP assay was performed by using anti-flag antibody. It was found that the OLD of p53 shows a higher binding efficiency to circ_CEA, compared to other domains of p53, suggesting that OLD of p53 may be important for the interaction between p53 and circ_CEA (Fig. 3H).

To investigate whether circ_CEA affects the association between p53 and CDK1, co-IP assay was conducted in 293 T cells transfected with circ_CEA expression vector or siRNA. p53 proteins co-immunoprecipitated by anti-CDK1 antibody were increased in the cells with circ_CEA overexpression, while the p53 proteins were decreased in the siRNA-transfected cells (Fig. 3I). These results suggest that circ_CEA promotes the interaction between CDK1 and p53 proteins.

### Circ_CEA promotes CDK1-mediated p53 phosphorylation at Ser315 and suppresses p53 activity in GC

**Fig. 4:**
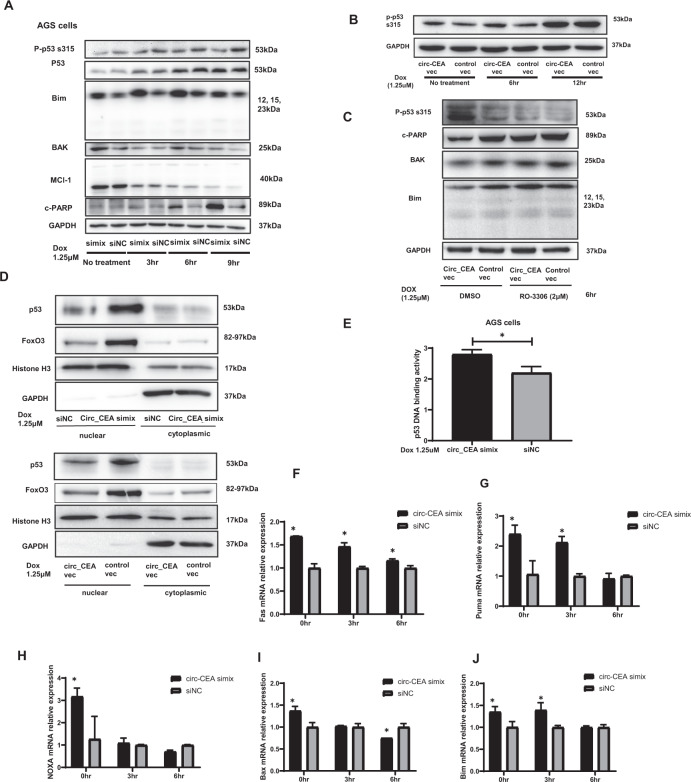
circ_CEA promotes CDK1-mediated p53 phosphorylation at Ser315 and suppresses p53 activity in GC. **A** AGS cells transfected with circ_CEA simix or siNC were treated with Dox (1.25 μM) for the indicated time. The expression levels of apoptosis-associated proteins were evaluated by western blotting assay. **B** AGS cells transfected with circ_CEA expression vector or control vector were treated with Dox (1.25 μM) for the indicated time, and the level of p53 phosphorylation at ser315 was evaluated by western blotting assay. **C** AGS cells transfected with circ_CEA expression vector or control vector were treated with Dox (1.25 μM) for 6 hr, in the presence or absence of RO-3306 (2 μM), a selective inhibitor of CDK1. Then the levels of apoptosis-associated proteins were evaluated by western blotting. DMSO was used as a vehicle control for Ro-3306. **D** AGS cells transfected with circ_CEA simix or siNC were treated with Dox (1.25 μM) for 2 hr (upper). In addition, AGS cells transfected with circ_CEA or control vectors were treated with Dox (1.25 μM) for 2 hr (lower). Then nuclear and cytoplasmic proteins were isolated, respectively. The levels of p53 and FoxO3 in nuclear and cytoplasmic fractions were determined by Western Blotting assay, respectively. Histone H3 and GAPDH were used as positive controls in nuclear and cytoplasmic fractions, respectively. **E** After Dox treatment (1.25 μM) for 1 hr, p53 DNA binding activity was evaluated in AGS cells transfected with circ_CEA simix or siNC. **F**–**J** AGS cells were transfected with circ_CEA simix or siNC, followed by Dox treatment for indicated time. The expression levels of p53 target genes (Fas, Puma, NOXA and Bax) and FoxO3 target gene, Bim were evaluated by qRT-PCR assay. *P < 0.05 vs corresponding siNC-transfected cells. Data are represented as mean± standard deviation (SD).

CDK1-induced p53 phosphorylation at ser315 reduces p53 stability and suppresses p53 functions [45, 47]. As we have shown that circ_CEA promotes the interaction between CDK1 and p53 proteins, we investigated whether circ_CEA affects p53 phosphorylation at ser315. siRNA-transfected AGS cells were treated with Dox (1.25 μM) for the indicated time, then the levels of apoptosis-associated proteins were determined by Western Blotting. Circ_CEA downregulation significantly decreased p-p53 ser315 level, but did not affect the total p53 level (Fig. 4A). Circ_CEA knockdown increased the levels of pro-apoptotic Bim and Bcl-2 homologous antagonist/killer (BAK), but did not affect anti-apoptotic myeloid cell leukemia-1 (Mcl-1) protein level significantly (Fig. 4A). The c-PARP level was increased in siRNA-transfected cells, indicating the enhancement of apoptosis (Fig. 4A). Circ_CEA overexpression increased p-p53 ser315 level in AGS cells (Fig. 4B). These findings suggested that circ_CEA promotes p53 phosphorylation at ser315 and suppresses the expression of pro-apoptotic proteins. To further confirm that circ_CEA promotes CDK1-mediated p53 phosphorylation at ser315, RO-3306, a selective CDK1 inhibitor was used. AGS cells transfected with circ_CEA or control vector were treated with Dox for 6 hr, in the presence or absence of RO-3306. Then, Western Blotting was conducted to evaluate the expression of apoptosis-associated proteins. Circ_CEA overexpression significantly increased p-p53 s315 level, while this increase was suppressed by RO-3306 treatment (Fig. 4C). c-PARP level was decreased in the cells with circ_CEA overexpression, indicating that circ_CEA inhibits apoptosis, while this decrease was restored by RO-3306 treatment (Fig. 4C). Additionally, the decrease in pro-apoptotic BAK and Bim induced by circ_CEA overexpression was restored by RO-3306 treatment (Fig. 4C). These results suggest that circ_CEA enhances CDK1-mediated p53 phosphorylation at s315, suppressing pro-apoptotic protein expression, and this leads to inhibition of apoptosis.

We next investigated the effect of circ_CEA on p53 activity. After Dox treatment, increased nuclear levels of p53 and FoxO3 were observed in circ_CEA siRNA-transfected cells. While the nuclear levels of p53 and FoxO3 were decreased in the cells with circ_CEA over-expression. These results suggested that circ_CEA suppresses the nuclear retention of p53 and FoxO3 (Fig. 4D). Furthermore, circ_CEA downregulation significantly increased p53 DNA binding activity, after Dox treatment (Fig. 4E). siRNA-transfected AGS cells were treated with Dox for the indicated time, and then the expression levels of p53 target genes (Fas, Puma, NOXA, Bax), and FoxO3 target gene (Bim) were determined by qRT-PCR assay (Fig. 4F–J). Circ_CEA downregulation markedly increased the expression of these p53 and FoxO3 target genes (Fig. 4F–J). These findings indicate that after Dox treatment, circ_CEA suppresses p53 nuclear retention and DNA binding activity, and further inhibits the expression of p53 target genes.

### circ_CEA downregulation suppresses tumor growth and promotes apoptosis in vivo

**Fig. 5:**
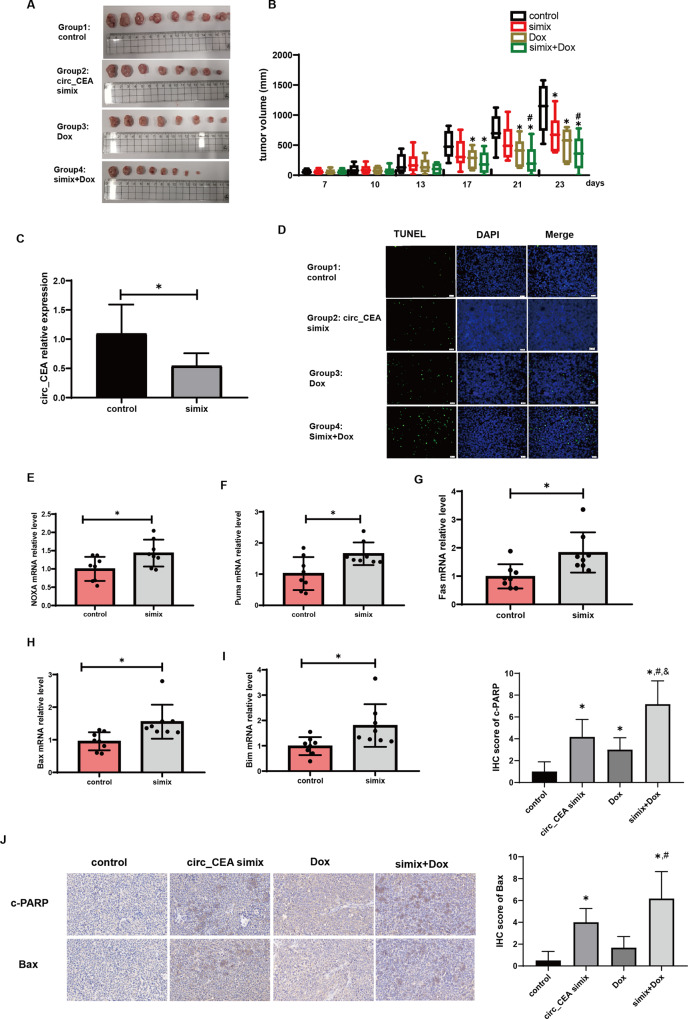
circ_CEA downregulation suppresses tumor growth and promotes apoptosis in vivo. **A** photos of subcutaneous tumors. After subcutaneous injection of AGS cells, BALB/c nude mice were randomly divided into four groups, and administrated with siNC (control group), circ_CEA simix, Dox+siNC (Dox group), and circ_CEA simix + Dox, respectively. **B** The tumor sizes were measured twice a week. *P < 0.05 vs control group, ^#^*P* < 0.05 vs circ_CEA simix group. **C** The expression level of circ_CEA in subcutaneous tumors was evaluated by qRT-PCR assay. **D** Paraffin sections were prepared from the subcutaneous tumors, and TUNEL assay were performed to evaluate apoptosis. **E**–**I** The expression levels of p53 target genes (NOXA, Puma, Fas, and Bax) and FoxO3 target gene, Bim in the subcutaneous tumors were determined by qRT-PCR assay. **J** IHC of c-PARP and Bax in the xenograft tumors from nude mice. *P < 0.05 vs control group; ^#^*P* < 0.05 vs circ_CEA simix group; ^&^*P* < 0.05 vs Dox group.

To investigate the effect of circ_CEA on tumor growth in vivo, we generated subcutaneous xenografts by injecting AGS cells into BALB/c nude mice. These mice were then randomly divided into 4 groups: 1. control, 2. circ_CEA simix, 3. Dox and 4. simix+Dox. Compared to the control group, circ_CEA simix, or Dox administration alone suppress tumor growth, while the inhibitory effect of their combined administration was significantly more marked (Fig. 5A, B). qRT-PCR assay confirmed that circ_CEA expression was significantly suppressed in vivo, following circ_CEA siRNA administration (Fig. 5C). Next, TUNEL assay showed that the administration of circ_CEA siRNA or Dox alone induced moderate apoptosis in the subcutaneous tumor xenografts, while the combined administration further enhanced apoptosis (Fig. 5D). qRT-PCR assay showed that circ_CEA simix administration induced significant upregulation of p53 pro-apoptotic target genes NOXA, Puma, Fas, Bax, and the FoxO3 target gene, Bim (Fig. 5E–I). These findings suggested that circ_CEA knockdown suppresses tumor growth, and promotes apoptosis via regulating the expression of p53 target genes in vivo. The combined administration of circ_CEA siRNA and Dox showed synergistic suppressive effects on tumor growth and promotive effects on apoptosis. In addition, IHC assay was performed to determine the expression levels of pro-apoptotic proteins, Bax and c-PARP, in subcutaneous xenografts (Fig. 5J). Compared to the control group, the administration of circ_CEA siRNA or Dox alone increased the levels of these pro-apoptotic proteins. While the upregulation of these proteins was more obvious in the group of combined administration of circ_CEA siRNA and Dox.

### circ_CEA downregulation suppresses lung metastasis of GC in vivo

**Fig. 6:**
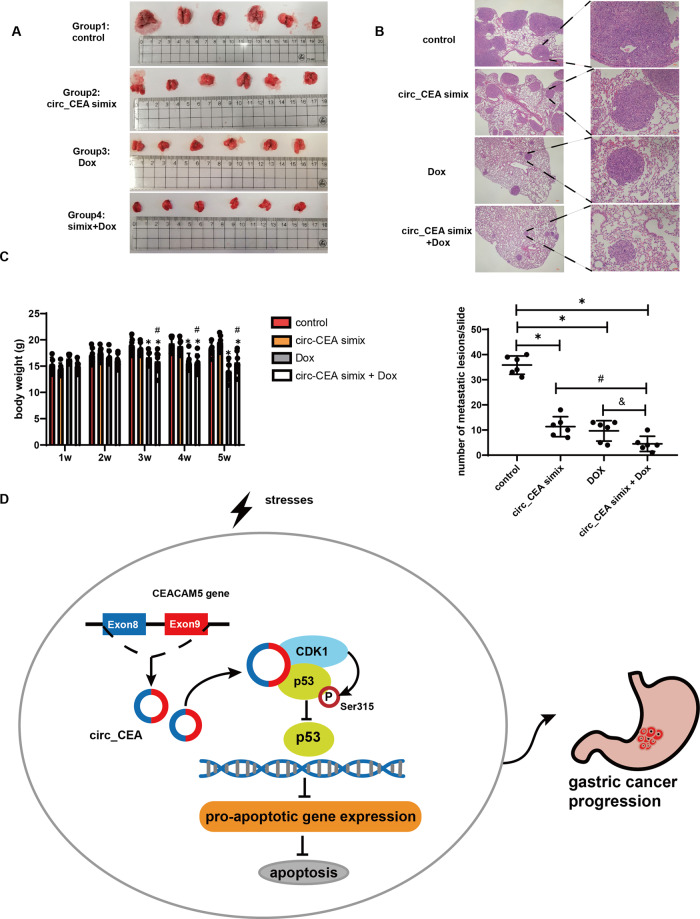
circ_CEA downregulation suppresses lung metastasis of GC in vivo. **A** The photos of mouse lungs. After tail vein injection of AGS cells, BALB/c nude mice were randomly divided into four groups, and administrated with siNC (control group), circ_CEA simix, Dox +siNC (Dox group), and circ_CEA simix + Dox, respectively. **B** the representative images of lung metastatic focuses (HE staining, upper). The metastatic focuses were quantified (lower). *P < 0.05 vs control group, ^#^*P* < 0.05 vs circ_CEA simix group, ^&^*P* < 0.05 vs Dox group. **C** The body weights of the mice were measured twice a week. *P < 0.05 vs control group; ^#^*P* < 0.05 vs circ_CEA simix group. **D** The speculated mechanism underlying the inhibitory effect of circ_CEA on stress-induced apoptosis in GC.

To investigate the effect of circ_CEA on tumor metastasis, we generated a metastasis model of GC via tail vein injection of AGS cells into BALB/c nude mice. The mice were randomly divided into 4 groups:1. control, 2. circ_CEA simix, 3. Dox, and 4. simix+Dox. Severe lung metastasis (the enlargement of lungs and massive metastatic lesions) was noted in the control group (Fig. 6A, B). Compared to the control group, Dox or circ_CEA simix administration alone significantly decreased the metastatic lesions in the lungs (Fig. 6B). Combined administration exhibited significantly larger suppressive effects on lung metastasis compared to Dox or circ_CEA simix administration alone (Fig. 6B). Obvious liver metastasis was not observed in any experimental group (Supplementary Fig. 3D). Additionally, the mice administrated with Dox or Dox + simix showed body weights that were significantly lower, compared to mice in the control or circ_CEA simix group (Fig. 6C). There was no significant difference in mouse body weight between control and circ_CEA simix group, suggesting that circ_CEA simix administration did not induce severe side-effects (Fig. 6C). Chemotherapy toxicity is a major concern when treating cancer patients. The reduced toxicity and enhanced therapeutic effect can be achieved using combinational treatment of chemo-drugs and circ_CEA siRNA. This has significant translational application meaning. Our findings showed that circ_CEA downregulation reduces lung metastasis in vivo. The combined administration of circ_CEA siRNA and Dox showed synergistic suppressive effects on tumor metastasis.

## Discussion

CircRNAs play critical roles in tumorigenesis and progression. Here, we showed that circ_CEA is upregulated in GC tissues and cell lines, and impacts stress-induced apoptotic response, with implications for metastasis and tumor growth. Mechanistically, we first report that circ_CEA promotes GC progression via acting as a protein scaffold to promote p53 phosphorylation.

Recently, the dysregulation of circRNAs in the development of various cancer has received more and more attention. Wang et al. have investigated circRNA expression profiles during cancer progression and identified 1209 dysregulated circRNAs in various types of cancer [48]. That circ_CEA (circBase ID: hsa_circ_0051240) was not included in the 1209 circRNAs may attribute to the heterogeneity of gastric carcinoma. Interestingly, Wang et al. showed that compared to other cancer types, GC had the least dysregulated circRNAs. In addition, they also showed the downregulation of global circRNA abundance in GC. Therefore, identifying an upregulated circRNA in GC (such as circ_CEA) may be important for the study of GC development and treatment. Several biological functions of circRNAs have been described [9]. Many studies on circRNA function show that circRNAs can act as miRNA sponges and regulate miRNA-mediated gene silencing [49–51]. Some circRNAs containing internal ribosome entry sites encode new proteins or peptides [52, 53]. In addition, some circRNAs interact with RNA binding proteins to regulate gene expression [54]. has_circ_0051240 (circ_CEA) is reportedly upregulated in ovarian cancer, and it exerts oncogenic functions via acting as a sponge of miR-637 [55]. Having identified circ_CEA as upregulated in GC, we sought to investigate the mechanisms by which it was potentially acting. Here, we demonstrated that circ_CEA enhances GC progression in vitro and in vivo. Particularly, we noted that it suppressed stress-induced apoptosis. As such we further investigated the mechanism by which circ_CEA regulates cellular apoptosis.

We hypothesized that circ_CEA regulates apoptosis via interacting with apoptosis-associated proteins. We identified the potential circ_CEA-binding proteins and demonstrated that circ_CEA interacts with CDK1 and its substrate p53, suggesting that circ_CEA may act as a scaffold to affect the association between p53 and CDK1. Some circRNAs have been reported to function as protein scaffolds and interact with enzymes and their substrates to promote their colocalization and interaction [56–58]. In this way, these circRNAs participate in regulation of protein ubiquitination [25, 26, 58, 59] and phosphorylation [57]. For example, it has been shown that circ_FoxO3 interacts with p53 and MDM2, and enhances MDM2-mediated p53 ubiquitination via promoting the binding of p53 to MDM2 [58]. circNDUFB2 is also found to act as a scaffold to promote the association between TRIM25 and Insulin-like growth factor 2 mRNA-binding proteins (IGF2BPs), and therefore enhance ubiquitination and degradation of IGF2BPs [26]. In addition, circ-Amotl1 binds to phosphoinositide dependent kinase and its substrate AKT1, resulting in the phosphorylation and nuclear localization of AKT1 [57]. Here, we showed that circ_CEA increased the level of p53 proteins which were co-immunoprecipitated by anti-CDK1 antibody, suggesting that circ_CEA facilitate the association between CDK1 and p53. It has been reported that CDK1 induces p53 phosphorylation at ser315 [45]. We further showed the promoting role of circ_CEA on p53 phosphorylation at ser315. Therefore, we suggest that circ-CEA enhances p53 phosphorylation at ser315, probably via acting as a protein scaffold and promoting the interaction between p53 and CDK1.

The phosphorylation of p53 at ser315 by several kinases plays an essential role in the regulation of p53 function [45, 60–62]. We showed that the increase in p-p53 s315 level and the decrease in pro-apoptotic proteins induced by circ_CEA overexpression were restored by a selective CDK1 inhibitor, RO-3306, suggesting that circ_CEA promotes CDK1-mediated p53 phosphorylation at ser315. Furtherly, it has been reported that CDK1-mediated p53 phosphorylation at ser315 negatively regulate p53 stability and function [47], and that cervical carcinoma cells became more susceptible to camptothecin, an anti-tumor agent, when cyclin B1, the regulatory subunit of CDK1, was silenced. In our study, circ_CEA suppressed the nuclear retention of p53. Circ_CEA knockdown increased DNA binding activity of p53 in response to Dox treatment, and further induced the upregulation of p53 pro-apoptotic target genes. These findings suggested that circ_CEA protects GC cells from Dox-induced apoptosis, via promotion of CDK1-mediated p53 phosphorylation at ser315 and further suppression of p53 activity.

We also found that circ_CEA also interacts with FoxO3 protein. FoxO3 regulates cellular stress responses upon various events, including shortage of nutrition and DNA damage [44]. FoxO3 participates in cellular apoptosis by regulating the expression of apoptosis-associated genes, including the pro-apoptotic Bim. Here, we showed that circ_CEA downregulation increased nuclear retention of FoxO3 and Bim expression, following Dox treatment. Therefore, circ_CEA suppresses Dox-induced apoptosis, partially through interacting with FoxO3 and suppressing its activity.

Translating these mechanistic findings to a clinically relevant model, we found that circ_CEA siRNA administration moderately suppressed tumor growth and lung metastasis, and induced apoptosis in vivo. The combined administration of circ_CEA siRNA and Dox exhibited synergistic anti-tumor effects in vivo, suggesting that the downregulation of circ_CEA renders GC cells more susceptible to Dox treatment. Furthermore, better therapeutic effects and less chemotherapy toxicity can be achieved by combinational treatment. This addresses a major concern for both patients and clinicians for the side-effects and long-term sequelae of anti-cancer chemotherapy. Therefore, the combination of Dox and circ_CEA silencing might be a strategy to enhance the anti-tumor effects and reduce side effect of Dox in GC.

## Conclusion

In summary, our study reveals a novel mechanism by which circ_CEA suppresses stress-induced apoptosis in GC (Fig. 6D). circ_CEA, which derives from the back-splicing of exon 8 and 9 of CEA gene, interacts with CDK1 and p53 proteins. It serves as a protein scaffold to enhance the interaction between CDK1 and p53, and therefore, promotes CDK1-mediated p53 phosphorylation at ser315. circ_CEA suppresses the nuclear retention and DNA binding activity of p53, leading to the downregulation of p53 pro-apoptotic target genes. Eventually, circ_CEA protects GC cells from stress-induced apoptosis and promotes GC progression. Therefore, our findings provide a potential future therapeutic target for GC patients.

## Supplementary information

Supplementary Table 1

Supplementary Table 2

Supplementary Figure

Supplementary legends

checklist

Original Data File

## Data Availability

The data generated in this study are available within the article and its Supplementary data files.
