# Supplementary material for: Circ_CEA promotes the interaction between the p53 and cyclin-dependent kinases 1 as a scaffold to inhibit the apoptosis of gastric cancer
Source: Cell Death Dis. 2022 Sep 27;13(9):827. doi: 10.1038/s41419-022-05254-1 (PMC9515085; doi:10.1038/s41419-022-05254-1)
Supplement: Supplementary file 1 — Supplementary Table 1 [file 41419_2022_5254_MOESM1_ESM.docx]

| Circ_CEA | F: 5’-CTCAGCTGGGGCCACTG-3’ |
| --- | --- |
|  | R: 5’-GTGTCCGGCCCATCAGTC-3’ |
| CEA | F: 5’-CAGAGCAGACAGCAGAGACC-3’ |
|  | R: 5’-TAGTGAGCTTGGCAGTGGTG-3’ |
| FAS | F: 5’-ACCCGGACCCAGAATACCAA-3’ |
|  | R: 5’-AAGAAGACAAAGCCACCCCA-3’ |
| NOXA | F: 5’-CCTGCAGGACTGTTCGTGTT-3’ |
|  | R: 5’-ACTTCCAGCTCCGCCGTA-3’ |
| PUMA | F: 5’-GATGAAATTTGGCATGGGGTCTG-3’ |
|  | R: 5’-GCTCCCTGGGGCCACAAAT-3’ |
| Bax | F: 5’-CAGGATGCGTCCACCAAGA-3’ |
|  | R: 5’-CCAGTTGAAGTTGCCGTCAGA-3’ |
| Bim | F: 5’-CTGACTCTGACTCTCGGACTG-3’ |
|  | R: 5’-ATTACCTTGTGGCTCTGTCTGT-3’ |
| 18s | F: 5’-ATCCTCAGTGAGTTCTCCCG-3’ |
|  | R: 5’-CTTTGCCATCACTGCCATTA-3’ |
| GAPDH | F: 5’-GAAGGTGAAGGTCGGAGTC-3’ |
|  | R: 5’-GAAGATGGTGATGGGATTTC-3’ |

Supplementary Table 1 Primers for qRT-PCR assay
