# Supplementary material for: Circ_CEA promotes the interaction between the p53 and cyclin-dependent kinases 1 as a scaffold to inhibit the apoptosis of gastric cancer
Source: Cell Death Dis. 2022 Sep 27;13(9):827. doi: 10.1038/s41419-022-05254-1 (PMC9515085; doi:10.1038/s41419-022-05254-1)
Supplement: Supplementary file 2 — Supplementary Table 2 [file 41419_2022_5254_MOESM2_ESM.docx]

Supplementary Table 2 potential protein partners of circ_CEA

**GeneSymbol GeneSymbol GeneSymbol**
 1 HNRNPK 32 FBL 63 YME1L1
 2 BRCC3 33 RPL13AP3 64 SERPINF2
 3 GIT2 34 GEMIN4 65 ATAD3B
 4 HSPD1 35 **TP53** 66 GATAD2A
 5 EEF1G 36 SQSTM1 67 ZNF281
 6 SMN1 37 TRIM65 68 HADHB
 7 EXOSC6 38 RPS26 69 GRN
 8 SFXN1 39 **CDK1** 70 BCR
 9 DBT 40 ILF2 71 HOXD13
 10 GIT1 41 RPS2 72 PRDX1
 11 MAGED4 42 EXOSC7 73 H2AFZ
 12 NADK 43 IGF2BP1 74 LOR
 13 BABAM2 44 PDK3 75 RPSA
 14 ATP5F1C 45 DDHD1 76 MLF2
 15 SNRPD2 46 PCGF1 77 ARF4
 16 ATP5MD 47 ARID3B 78 KPRP
 17 ERCC1 48 EWSR1 79 LRP1
 18 TOMM22 49 WRNIP1 80 RPL10L
 19 HRNR 50 RFC5 81 GTSE1
 20 SLC25A18 51 NDUFA4 82 WDR5
 21 P4HA1 52 RPL12 83 EIF3E
 22 EXOSC9 53 RPS14 84 FCRL6
 23 EIF3F 54 SNX18 85 ZNF518A
 24 SKA1 55 DDX5 86 MAD2L1
 25 HEATR5B 56 PPHLN1 87 GTF3C5
 26 RBM4B 57 SSR3 88 RUVBL2
 27 ZNF326 58 RPS27L 89 IGKV6D-21
 28 SLX4IP 59 RUVBL1 90 ATP5ME
 29 SKA2 60 TRIM25 91 PTAR1
 30 SSR4 61 ATP5MG 92 HBS1L
31 ABRAXAS1 62 H3F3C 93 RFC4
 94 KLF16
