# Supplementary figures and images for: Circ_CEA promotes the interaction between the p53 and cyclin-dependent kinases 1 as a scaffold to inhibit the apoptosis of gastric cancer
Source: Cell Death Dis. 2022 Sep 27;13(9):827. doi: 10.1038/s41419-022-05254-1 (PMC9515085; doi:10.1038/s41419-022-05254-1)

A

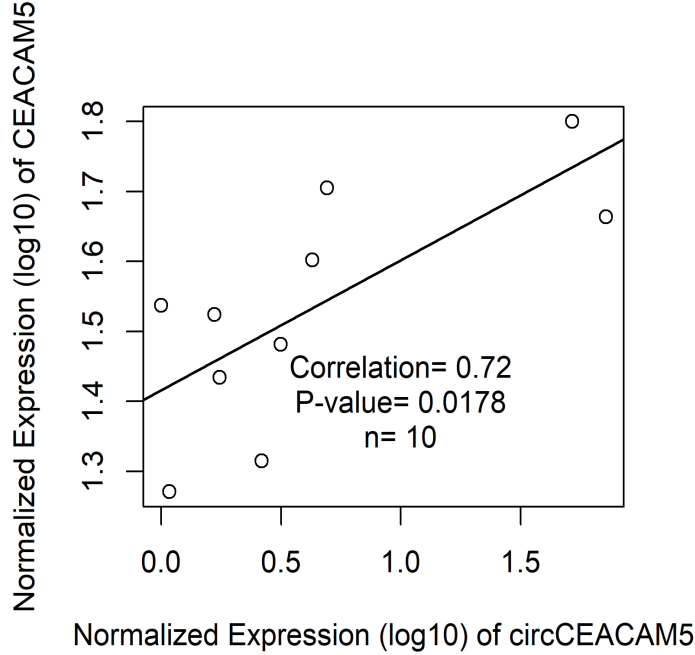

B

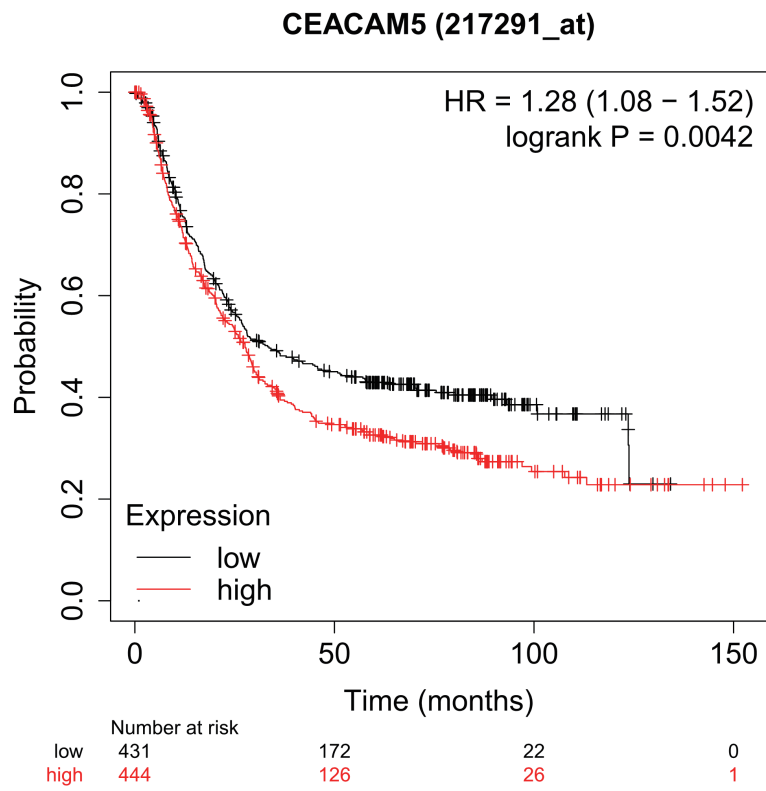

Supplementary figure 1

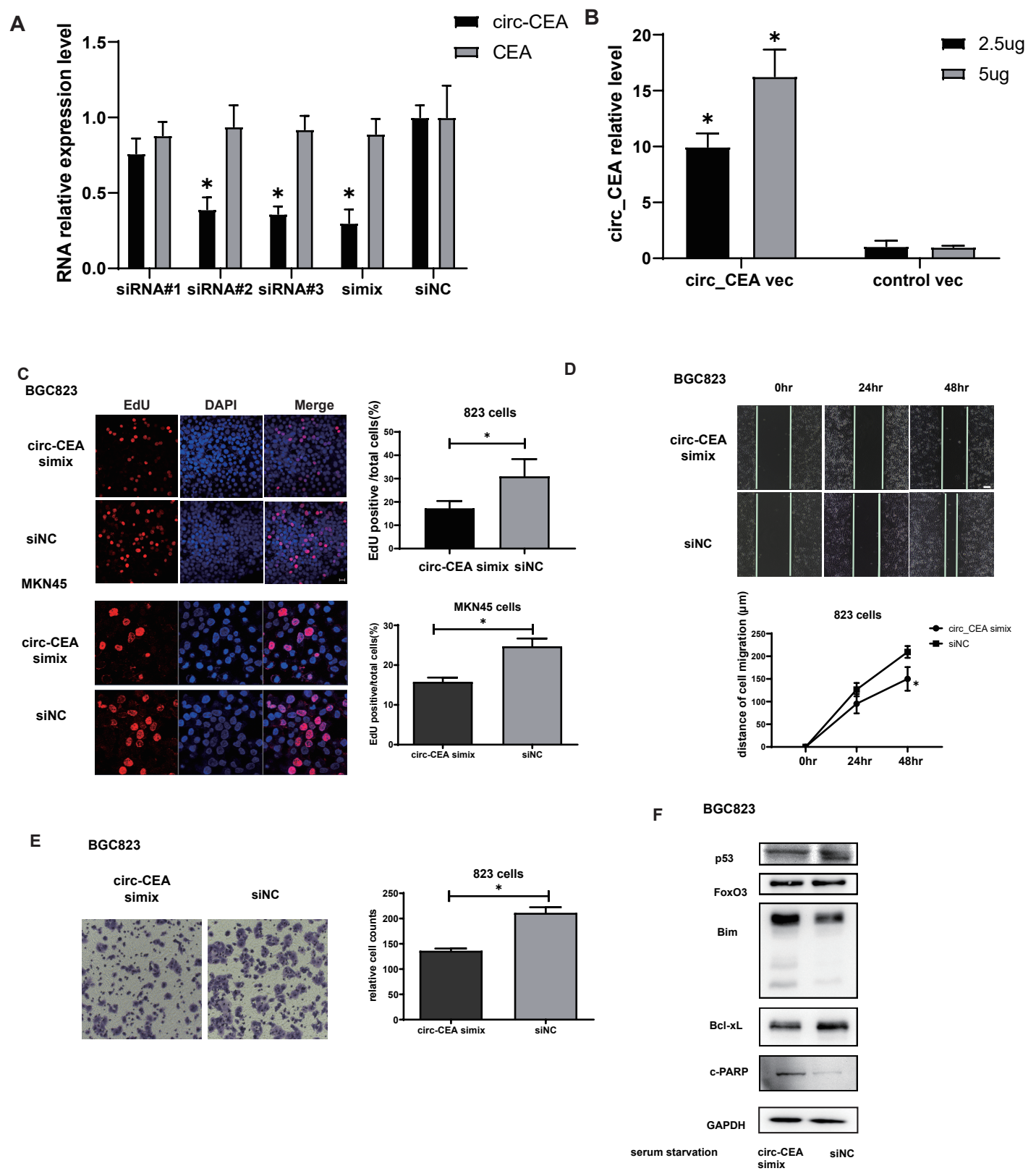

Supplementary figure 2

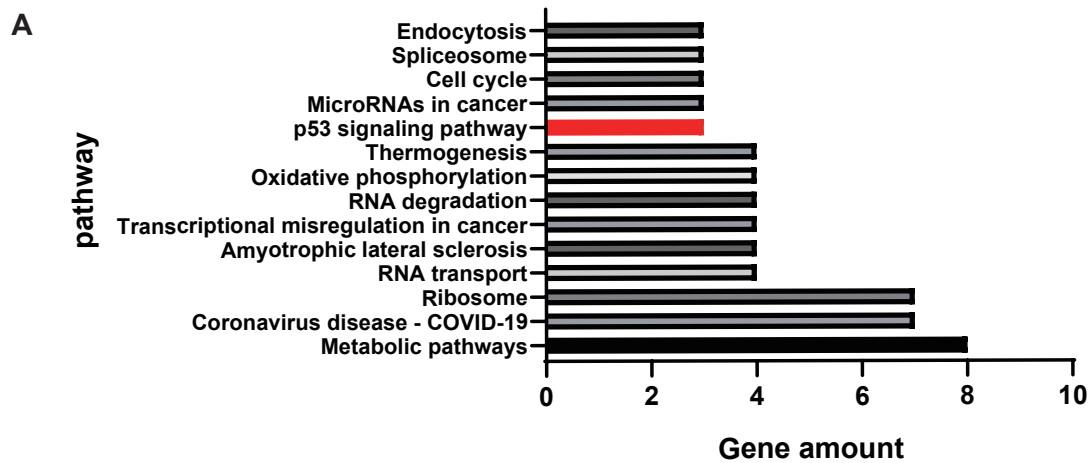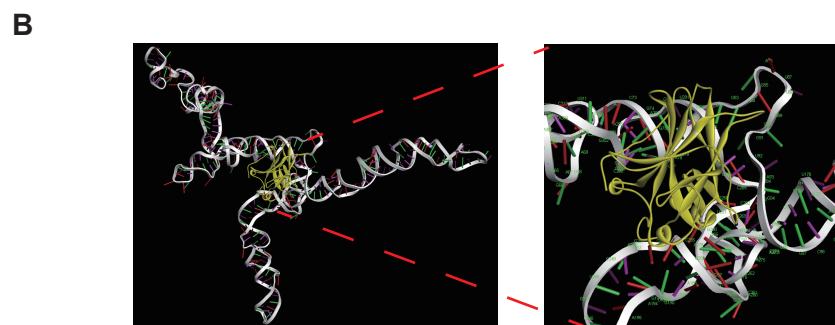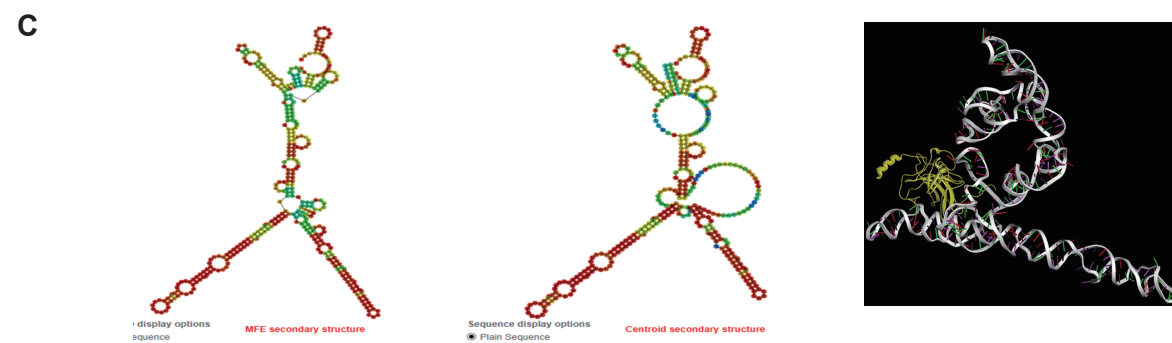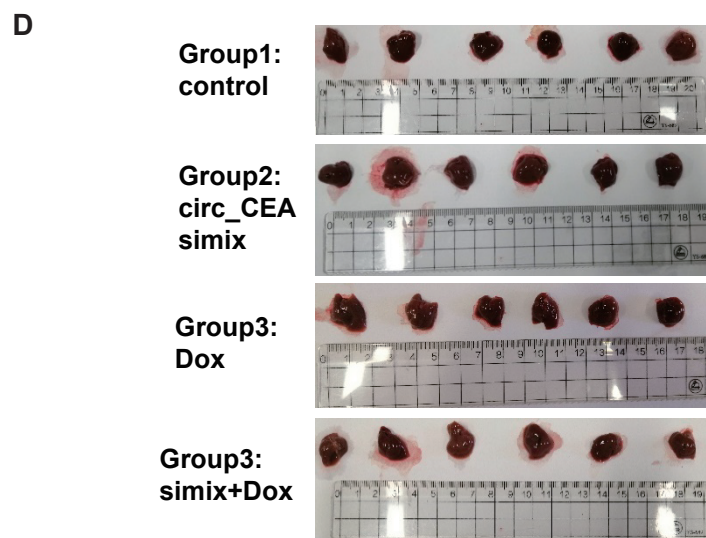

Supplementary figure 3

Supplement: Supplementary file 3 — Supplementary Figure [file 41419_2022_5254_MOESM3_ESM.pdf]
