# Supplementary material for: Circ_CEA promotes the interaction between the p53 and cyclin-dependent kinases 1 as a scaffold to inhibit the apoptosis of gastric cancer
Source: Cell Death Dis. 2022 Sep 27;13(9):827. doi: 10.1038/s41419-022-05254-1 (PMC9515085; doi:10.1038/s41419-022-05254-1)
Supplement: Supplementary file 4 — Supplementary legends [file 41419_2022_5254_MOESM4_ESM.docx]

**Supplementary legends**

**Supplementary Figure 1**

(A)The expression correlation analysis was performed between circ_CEA and CEA in 10 gastric tissues. (B) CEA expression is a poor prognostic factor for GC.

**Supplementary Figure 2**

(A) Three siRNAs (siRNA#1, siRNA#2, and siRNA#3) for circ_CEA were designed based on the sequence of its junction site. The levels of circ_CEA and linear CEA mRNA in AGS cells transfected with circ_CEA siRNAs or siNC were evaluated by qRT-PCR assay. Simix is the mixture of siRNA#2 and siRNA#3. *P<0.05 vs circ_CEA level in siNC-transfected AGS cells. (B). AGS cells were transfected with circ_CEA expression vector or control vector (vector 2.5 or 5 μg). qRT-PCR was performed to evaluate the expression of circ_CEA. *P<0.05 vs the circ_CEA level in corresponding control vector-transfected cells. (C) Cellular proliferation was evaluated by EdU assay in BGC-823 and MKN45 cells transfected with circ_CEA simix or siNC. (D) Cellular migration was determined via wound-healing assay in BGC-823 cells transfected with circ_CEA simix or siNC. (E) Cellular migration was further evaluated by transwell migration assay in BGC-823 cells transfected with circ_CEA simix or siNC. (F) The levels of apoptosis-associated proteins were evaluated by Western blotting in BGC-823 cells transfected with circ_CEA simix or siNC, after serum starvation treatment (72hr).

**Supplementary Figure 3**

(A) To identify potential binding proteins of circ_CEA, MS2/MS2-CP based RNA pull down and protein mass spectrometry was performed, followed by KEGG pathway enrichment analysis. (B) The interaction between circ_CEA and p53 was simulated in HDOCK website (http://hdock.phys.hust.edu.cn/), by using their sequences. Yellow line indicates p53 and white line indicates circ_CEA. (C)The secondary and tertiary structures of circ_CEA were predicted. The interaction between circ_CEA and p53 was simulated in HDOCK by using their tertiary structures. (D) Photos of the livers of mice injected with AGS cells via tail vain.
