# Supplementary material for: Circ_CEA promotes the interaction between the p53 and cyclin-dependent kinases 1 as a scaffold to inhibit the apoptosis of gastric cancer
Source: Cell Death Dis. 2022 Sep 27;13(9):827. doi: 10.1038/s41419-022-05254-1 (PMC9515085; doi:10.1038/s41419-022-05254-1)

Figure 2F

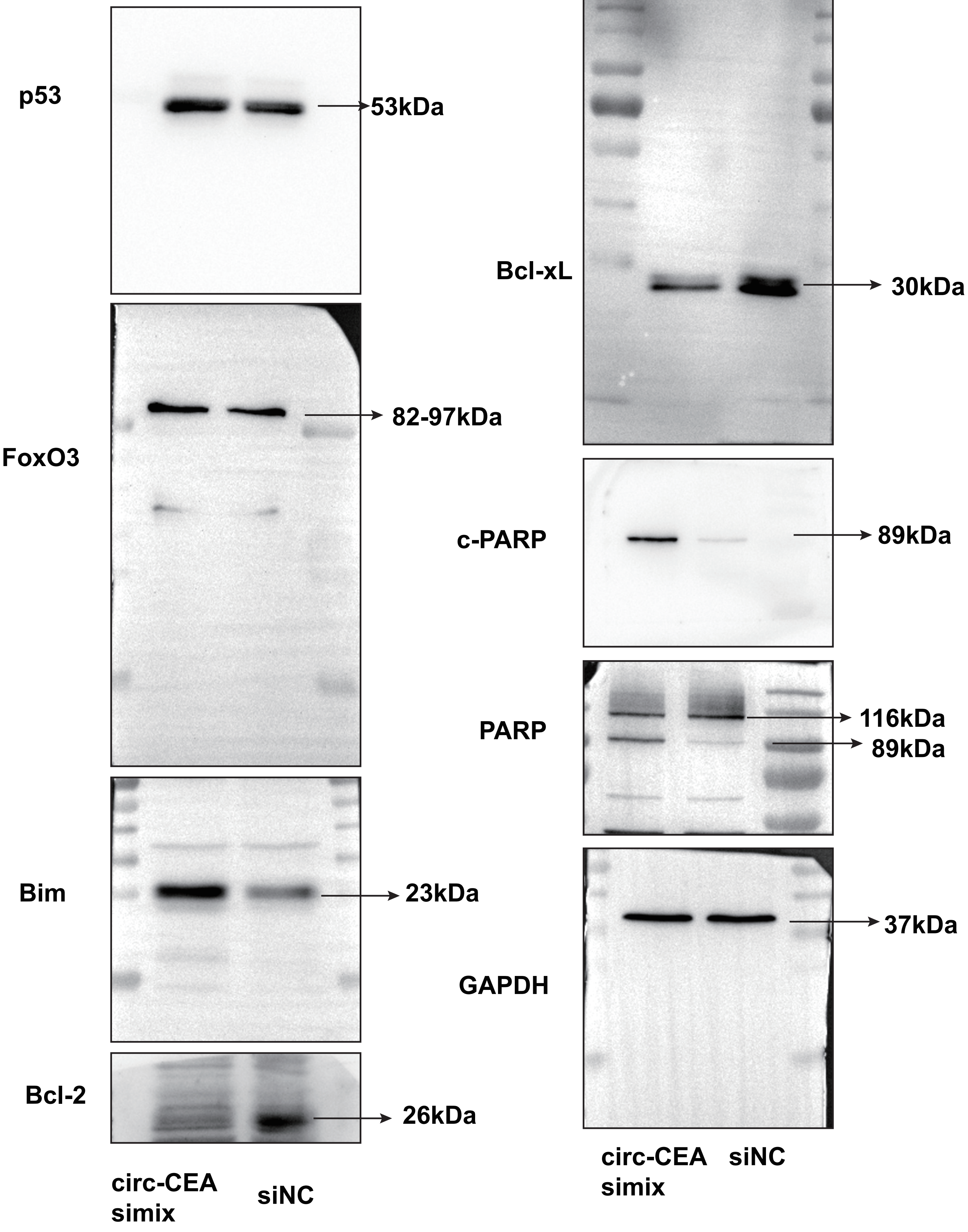

p53
53kDa
FoxO3
82-97kDa
Bim
23kDa
Bcl-2
26kDa
circ-CEA simix
siNC
Bcl-xL
30kDa
c-PARP
89kDa
PARP
116kDa
89kDa
GAPDH
37kDa
circ-CEA simix
siNC

Figure 2F

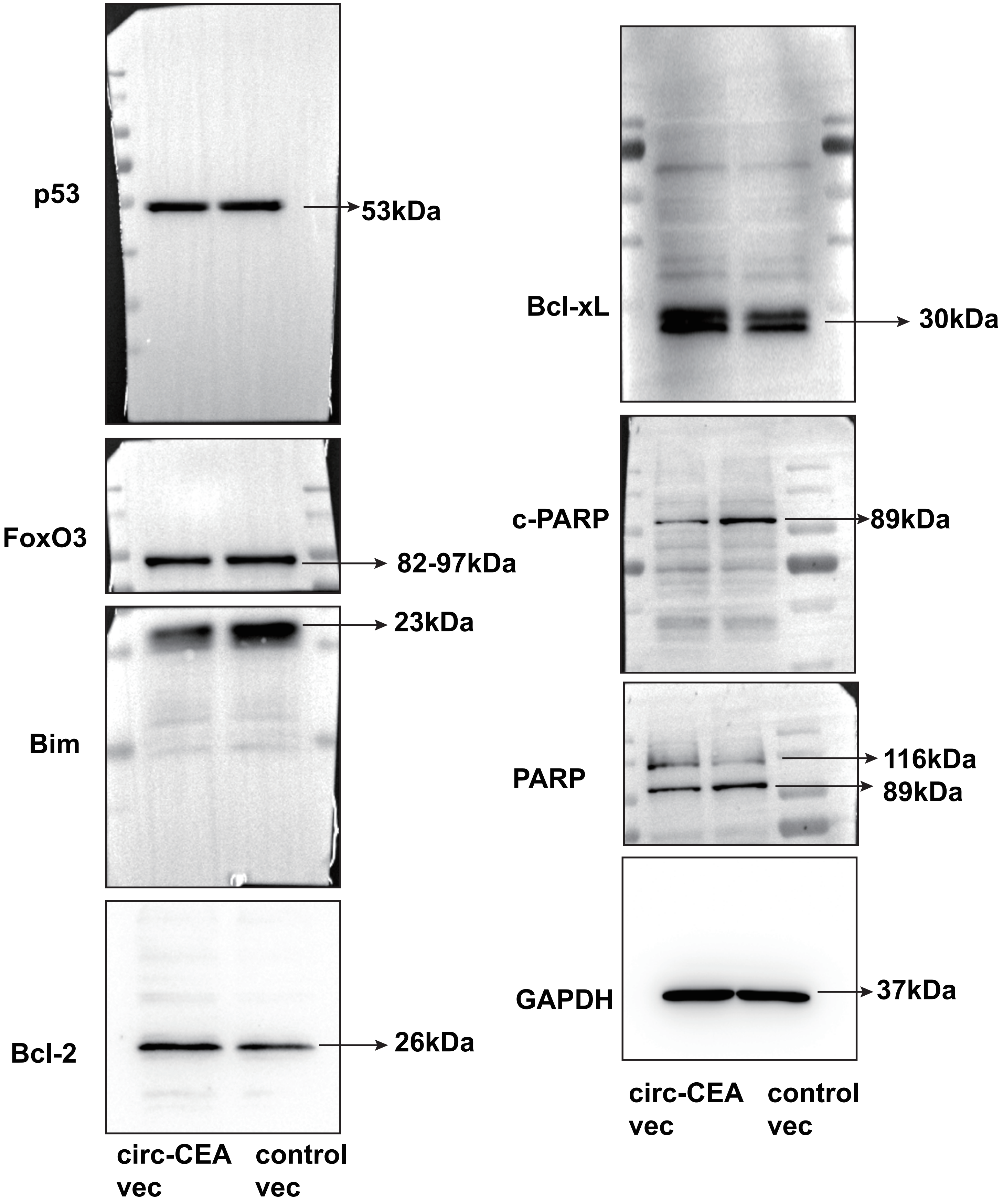
p53
53kDa
FoxO3
82-97kDa
Bim
23kDa
Bcl-2
26kDa
circ-CEA vec
control vec
Bcl-xL
30kDa
c-PARP
89kDa
PARP
116kDa
89kDa
GAPDH
37kDa
circ-CEA vec
control vec

Figure 3E

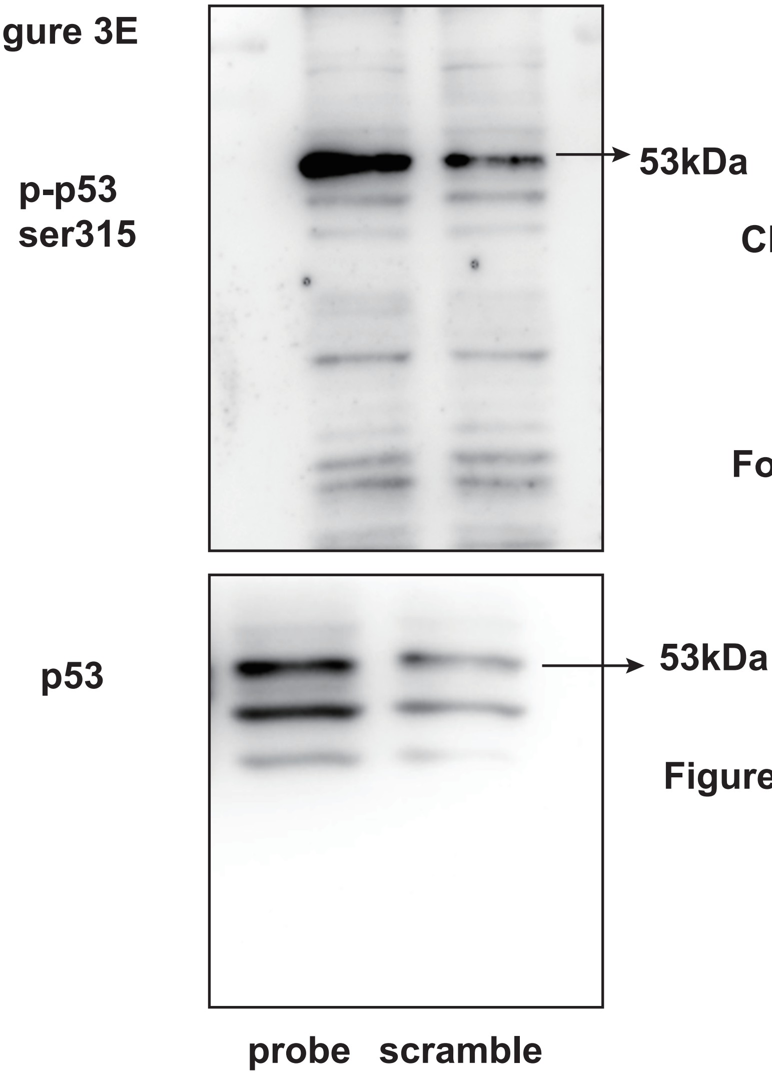

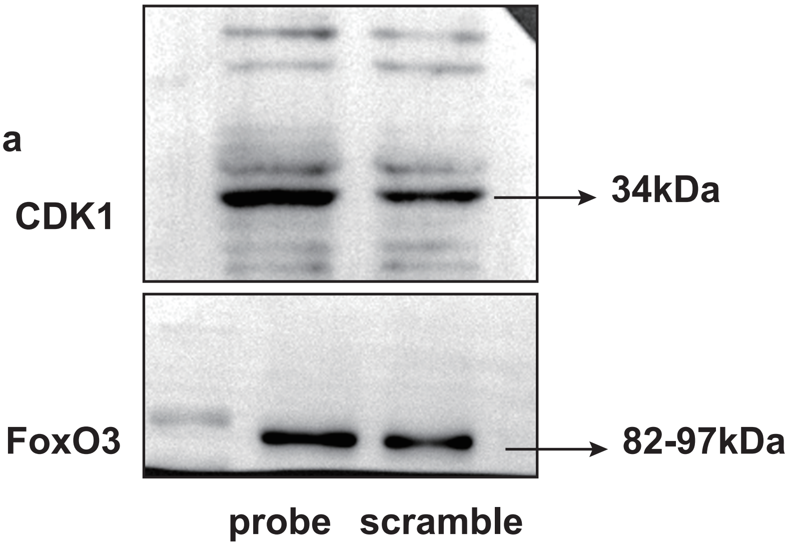

Figure 3H

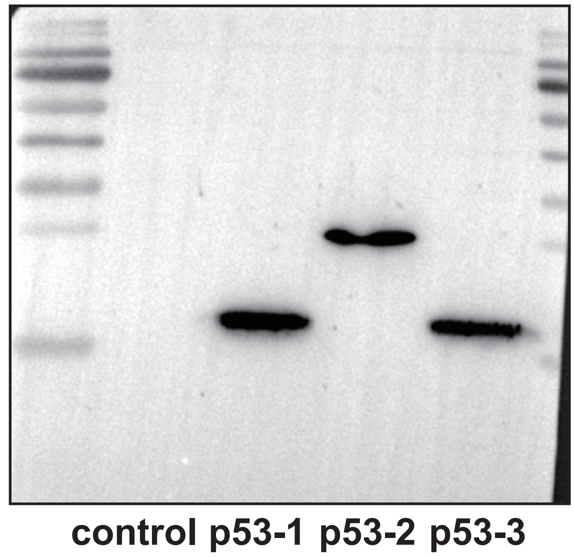

Figure 3I

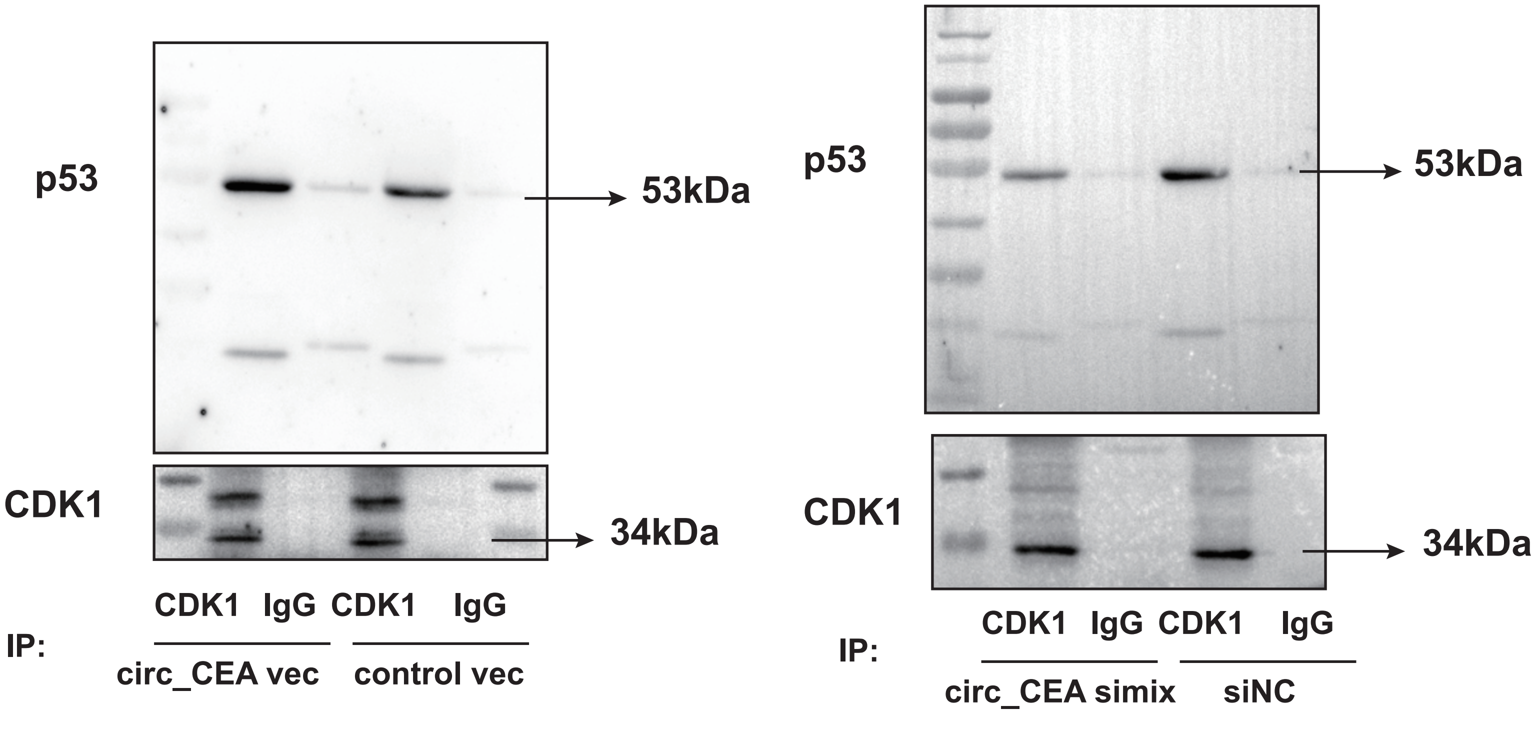

Figure 4A

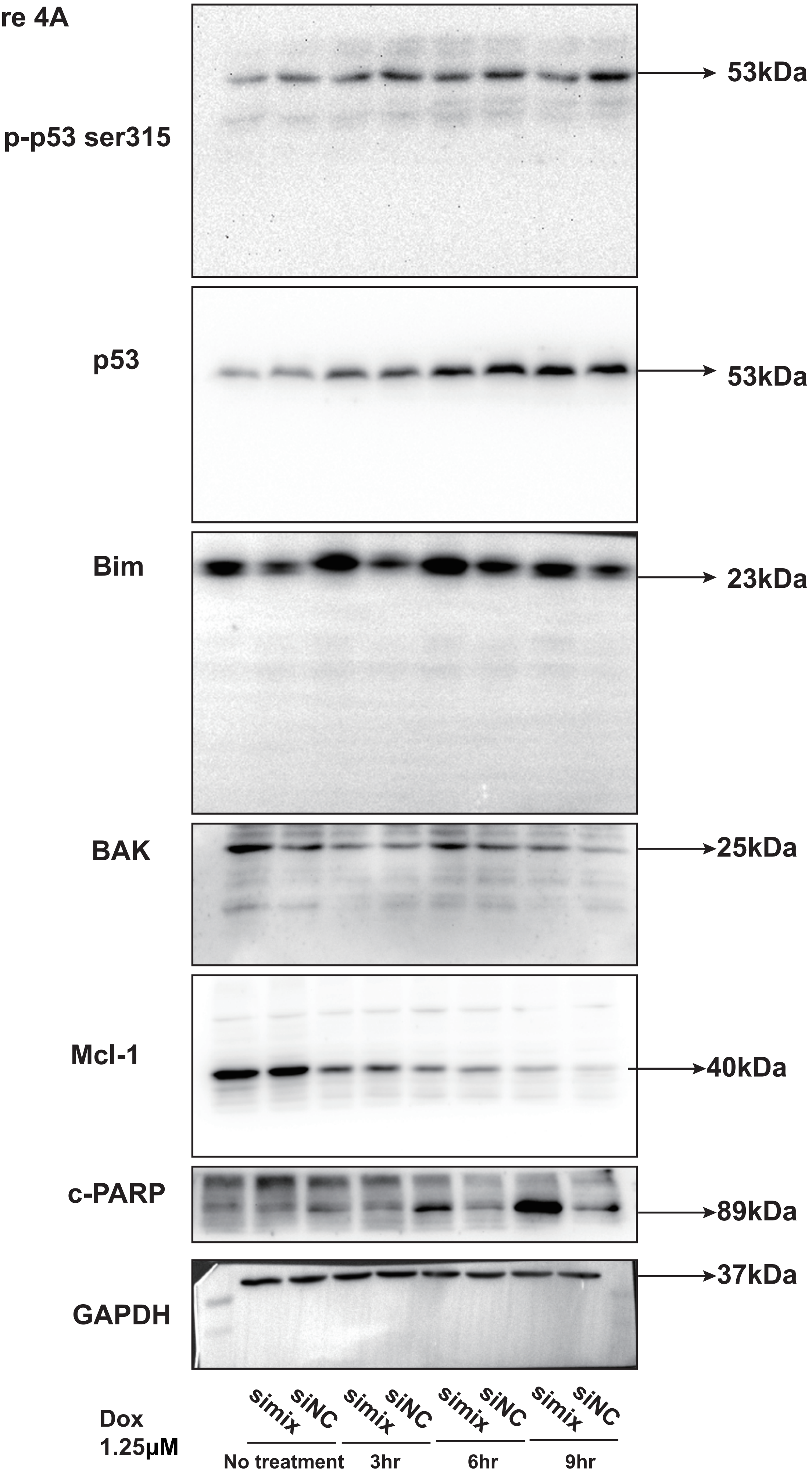

p-p53 ser315
53kDa
p53
53kDa
Bim
23kDa
BAK
25kDa
Mcl-1
40kDa
c-PARP
89kDa
GAPDH
37kDa
Dox
1.25μM
simix
siNC
simix
siNC
simix
siNC
simix
siNC
No treatment
3hr
6hr
9hr

Figure 4B

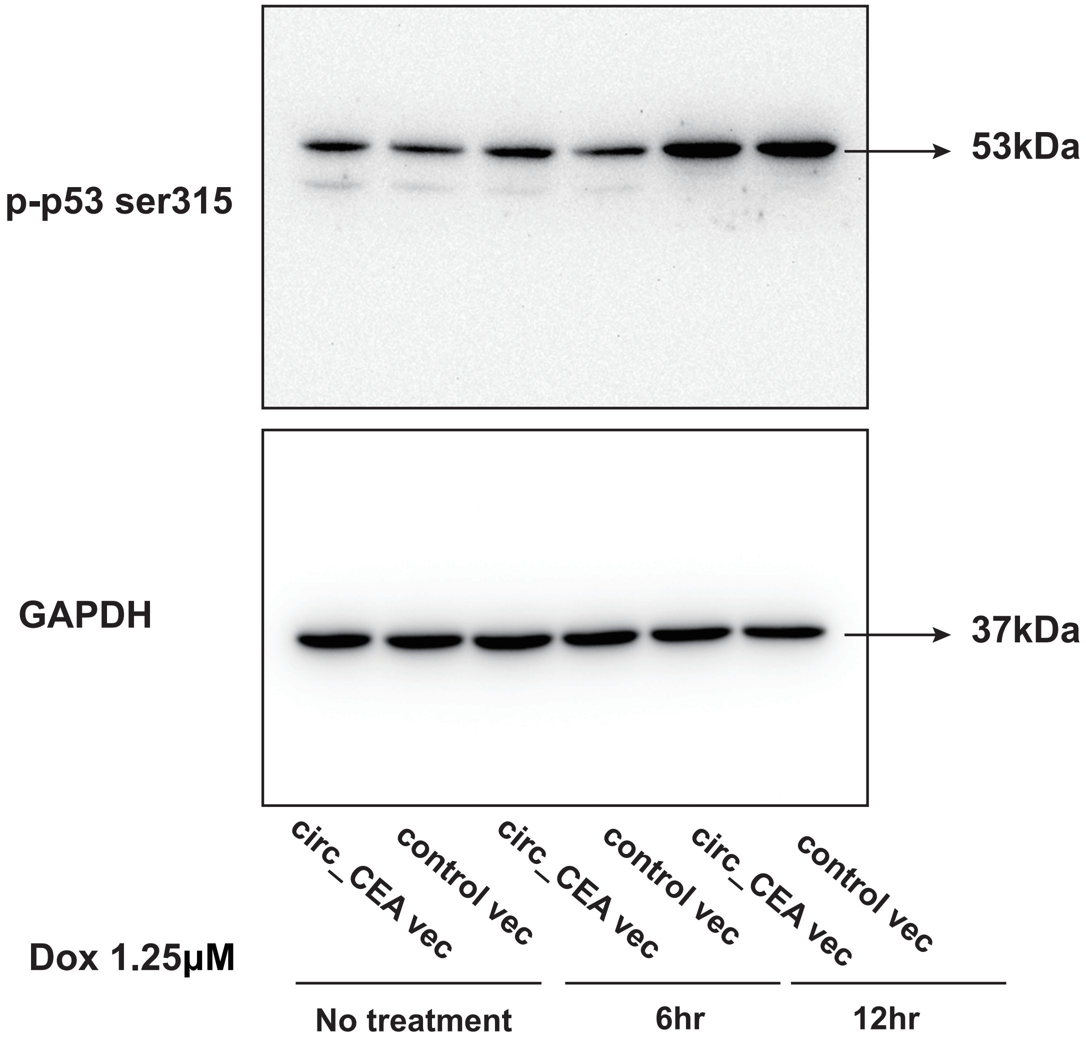

Figure 4C

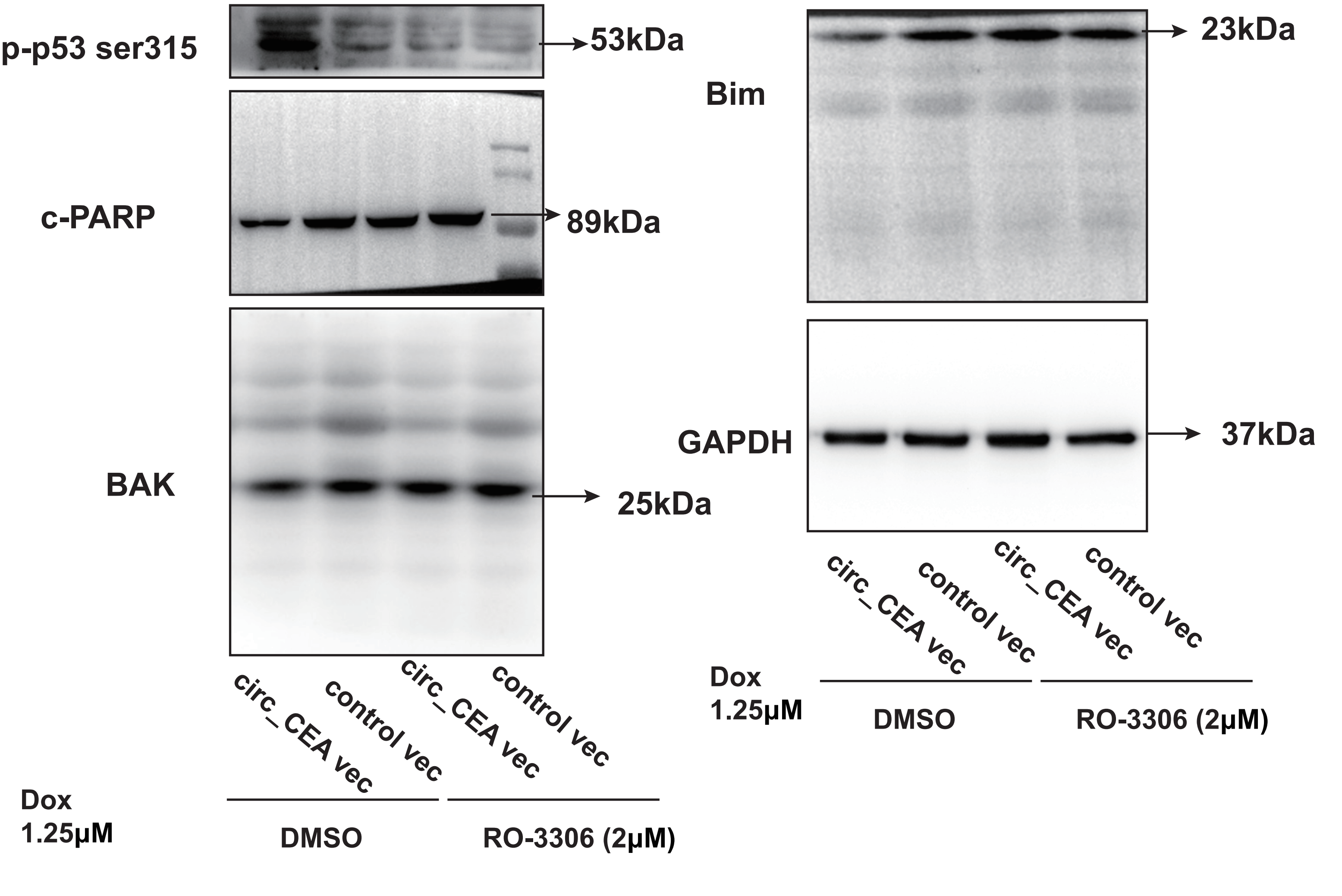

Figure 4D

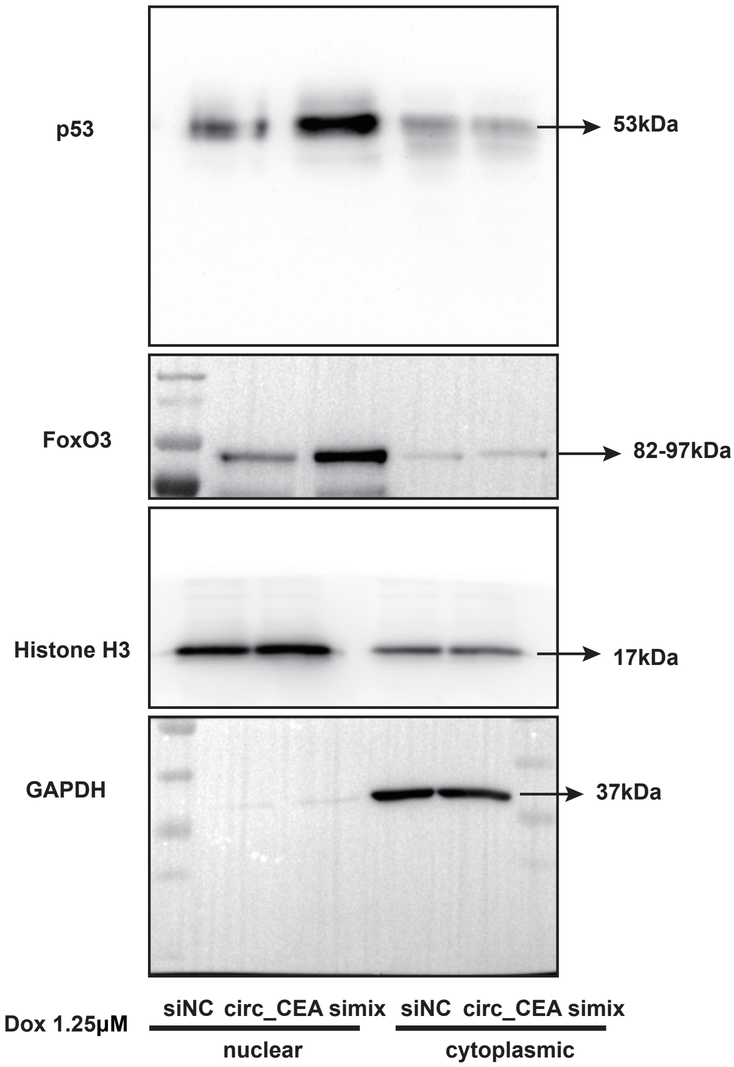

p53
53kDa
FoxO3
82-97kDa
Histone H3
17kDa
GAPDH
37kDa
Dox 1.25μM
siNC circ_CEA simix siNC circ_CEA simix
nuclear
cytoplasmic

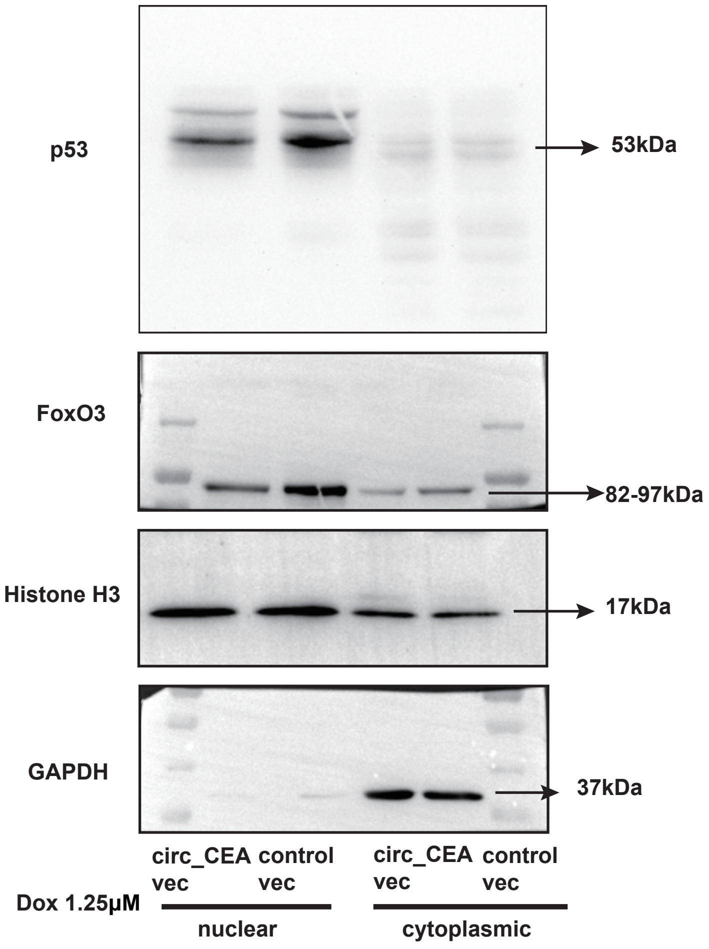

p53
53kDa
FoxO3
82-97kDa
Histone H3
17kDa
GAPDH
37kDa
circ_CEA vec
control vec
circ_CEA vec
control vec
Dox 1.25μM
nuclear
cytoplasmic

supplementary figure2

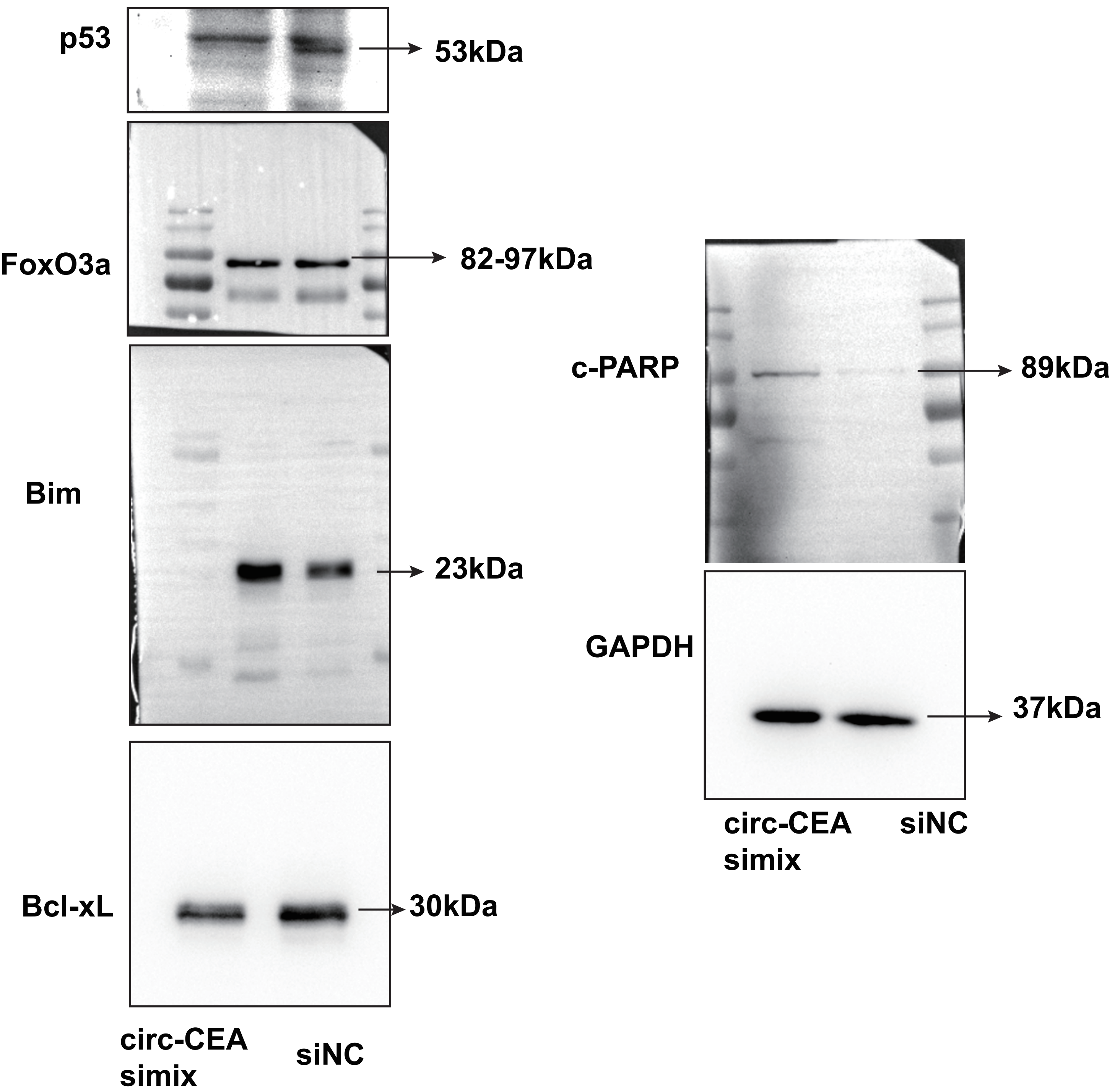

Supplement: Supplementary file 6 — Original Data File [file 41419_2022_5254_MOESM6_ESM.pdf]
